# Do hospital-to-home transitions work for older adults with multiple long-term conditions including dementia? A realist review

**DOI:** 10.1186/s12877-025-06123-0

**Published:** 2025-07-09

**Authors:** Lauren Lawson, Matthew Cooper, Clare Tolley, Annette Hand, Hamde Nazar

**Affiliations:** 1 National Institute for Health and Care Research Newcastle Patient Safety Research Collaboration, Newcastle University, Newcastle upon Tyne, UK; 2 School of Pharmacy, Newcastle University, Newcastle upon Tyne, UK; 3 University of Northumbria, Newcastle upon Tyne, UK; 4 Newcastle Hospitals NHS Foundation Trust, Newcastle upon Tyne, UK

**Keywords:** Multiple long-term conditions, Dementia, Hospital-to-home, Transitions of care, Realist review, Realist synthesis

## Abstract

**Background:**

Hospital-to-home transitions involve multiple providers and are particularly complex for older adults with dementia, who often live with additional conditions. Frequent transitions increase the risk of errors, miscommunication, and treatment delays, compromising patient safety and leading to potentially increased mortality, morbidity, and preventable readmissions. Understanding what works and does not work in these processes is essential to improving outcomes.

**Aim:**

This realist review synthesised existing literature to explore how, for whom, and to what extent hospital-to-home transitions work for older adults with multiple long-term conditions including dementia.

**Methods:**

Nine databases were systematically searched using key terms to identify evidence on hospital-to-home transitions for older adults (65+) with multiple long-term conditions including dementia. Interactions between contexts, mechanisms, and outcomes influencing transitions were identified and synthesised to develop a programme theory.

**Results:**

We included 68 peer-reviewed and 2 grey literature documents. Integral features of how transitions work were identified, including generic components of transitions, and five dementia-specific components which were the focus of this review: dementia care management, knowledge, information exchange standards, system features, and the role of friends/family. Fragmented care pathways and poor collaboration led to delays, unsafe discharges, and increased reliance on carers, exacerbating service gaps. Limited dementia training for providers and non-standardised documentation hindered effective discharge planning. Carers faced emotional distress and decision-making conflicts, often managing care responsibilities without adequate training, increasing risks of readmissions, particularly for unmanaged conditions.

**Conclusions:**

Hospital-to-home transitions are complex, requiring tailored interventions that address population-specific challenges. A realist approach provides valuable insights to inform development of relevant, supportive interventions in the future.

**Study registration:**

This review was preregistered with PROSPERO (CRD42023494003).

**Clinical trial number:**

Clinical trial number: not applicable.

**Supplementary Information:**

The online version contains supplementary material available at 10.1186/s12877-025-06123-0.

## Introduction

In the United Kingdom (UK), people with dementia occupy one in four hospital beds and face heightened risks as they return home from hospital, often encountering adverse outcomes that compromise safety [1, 2]. Dementia is characterised by progressive cognitive impairment that impacts daily activity, with prevalence in the UK projected to increase to over 2 million by 2051 [3, 4]. Older adults (aged ≥ 65 years) with dementia have approximately 4.6 additional, long-term conditions, resulting in complex care needs [5]. The co-existence of two or more chronic conditions in an individual, defined as multiple long-term conditions (MLTC), has placed additional strain on health and social care systems, which were designed to support single-disease management [6–9]. Consequently, older adults with multiple long-term conditions including dementia (MLTCiD) experience frequent transitions of care to access support and manage their conditions [10].

Transitions of care refers to patient movement between healthcare settings, or healthcare professionals (HCPs) to receive care [11]. Health and social care services in the UK are often described as fragmented, with longstanding policy efforts to improve service integration [9, 12]. Older adults with MLTCiD experience more transitions than those without dementia, which are more likely to involve fragmented care [13]. Lapses in patient safety during transitions, such as poor communication and lost test results during information transfer can delay appropriate treatment, increasing healthcare costs and service use [11]. Dementia-related hospital costs exceed £3.3 billion each year, driven by unplanned admissions, have doubled in the last decade [14, 15]. Research suggests that this population has a high risk of readmission, most of which are thought to be preventable and resulting from failures in continuity of care [16, 17].

Fragmented transitions can also lower physical and psychological wellbeing of those with MLTCiD and unpaid carers (referred to as ‘carers’) [18, 19]. Current guidelines and literature inadequately addresses the complexity of managing MLTCiD with a tendency for a single-disease focus (i.e., only one of 13 NICE guidance documents acknowledged the impact of dementia on care management [20]) [21–24]. To support transitions of care with MLTC, use of evidence-based models such as the Transitional Care Model have been well-documented, and adapted for people with dementia [10, 25]. The Transitional Care Model’s core principles of coordination, communication, collaboration, and continuity of care were identified as key to significant improvements in hospital-to-home transitions [25]. This emphasises the importance of improving these areas to support integrated approaches to managing MLTCiD, as highlighted by carers, healthcare and social care professionals [26, 27]. However, research suggests that a better understanding of which elements contribute to discharge-specific outcomes, such as readmission, preparation, and management at home is needed due to variations in which elements of the model have been included in practice [25].

Previous synthesis of quantitative evidence identified six elements influencing care transitions for this population: unmet needs, depression, education and support, physical decline, poor quality of health, and limited access to community services [28]. While these factors provide a broad framework, current evidence lacks clarity on the complexity of transitions, and the interplay between dementia and co-existing conditions, leaving gaps in knowledge about what works and why. Without wider consideration of available evidence it is unclear how these factors among others, and the context in which they occur, influence outcomes for older adults with MLTCiD allowing for the development of recommendations and improvements to current practice. The aim of this review was to explore how hospital-to-home transitions occur for older adults with MLTCiD. We aimed to understand how these transitions work (or not), for whom, and to what extent in order to develop and refine a programme theory of hospital-to-home transitions for this population and those involved in their care.

## Methods

A realist review is a theory-driven evidence synthesis method used to explain what works in an intervention, for whom, under which circumstances, and why [29]. It builds a programme theory based on assumptions of how an intervention works and its expected impacts, refined using empirical evidence [29, 30]. Causality is explained by examining the underlying mechanism (M) linking a context (C) to an outcome (O), forming context-mechanism-outcome configurations (CMOC) to iteratively refine the theory [29]. Results integrate the programme theory with evidence to explain how interventions work across different contexts [29]. Realist reviews are increasingly used in healthcare research as they can explore complex, context-dependent interventions, making this a suitable approach to understand highly variable hospital-to-home transitions for older adults with MLTCiD [31].

The realist approach followed processes set out by Pawson et al. and the Realist and Meta-narrative Evidence Synthesis: Evolving Standards (RAMESES) [29, 32]. This review was preregistered with PROSPERO (CRD42023494003) and has been reported in accordance with the Preferred Reporting Items for Systematic Reviews and Meta-Analyses (PRISMA) statement (see online supplementary materials) [33].

### Development of the initial programme theory

In the first stage of the review, an ‘initial programme theory’ (IPT) was developed based on an understanding of the processes involved in returning home from hospital, and barriers and enablers to their success. This was informed by existing knowledge and supported by informal searches of relevant literature, for example searching reference lists and using ‘cited-by’ searching functionality. Potentially relevant theories were discussed within the research team, and key processes were collated into the IPT.

### Searching and selection of documents

The search strategy, guided by keywords and relevant prior strategies, was piloted on MEDLINE and adapted as required (see supplementary materials). Searches were conducted across seven electronic databases (CINAHL, Embase, MEDLINE, PsycINFO, PubMed, Scopus, Web of Science) and two grey literature databases (Health Management Information Consortium, ProQuest Dissertations and Theses) between February 23rd − 28th, 2024. Reference lists of included documents were hand-searched, and ‘cited-by’ searching was undertaken to purposively identify additional relevant documents. No date restrictions were placed on the searches as evidence of how hospital-to-home transitions have worked in the past for this population may have improved understanding of the current relationships between contexts, mechanism and outcomes.

Search results were exported into a reference management software (Endnote) to remove duplicates. Remaining documents were then exported into Rayyan, an electronic software tool used to facilitate screening [34]. Screening was performed at title, abstract and keyword level, then at full-text by one reviewer (LL), with 10% checked by a second reviewer (MC, CT, HN) for consistency. A 20% sample of the documents for final inclusion were independently checked by three reviewers (MC, CT, HN), with disagreements resolved through discussion.

Included documents were published in English, focused on hospital-to-home transitions for older adults with any dementia and at least one other long-term condition, and/or carers, health, or social care professionals involved in their care. We did not include older adults living in aged care facilities that do not focus on independent living (e.g. nursing homes), or with a life expectancy under three months. The focus of this synthesis was older adults with MLTCiD returning to the community to self-manage their conditions, therefore these exclusion criteria were applied as these populations were very likely to have different outcomes after transitions that would not be relevant to our programme theory [35]. Documents were selected for inclusion based on their relevance to the IPT and included a diverse range of literature, such as commentaries or theoretical papers which could contribute to programme theory development [36]. Quality was assessment for relevance (i.e. contributed to IPT development/testing) and rigour (ensuring data were credible and trustworthy) [36].

### Data extraction and synthesis

Relevant characteristics (e.g., bibliographic details, study design, participant details, settings, findings) were extracted into an Excel spreadsheet. Full-text documents were uploaded into NVivo, a qualitative data analysis software tool, to facilitate organisation and coding of data. Coding of sections of the texts was inductive (emerging from the data), deductive (informed by the IPT), and retroductive (based on interpretation of data) [36]. Codes identified prominent contexts, mechanisms, and their relationship to outcomes in the data.

Analysis and synthesis occurred iteratively, guided by realist logic to refine the programme theory [36]. Data from each document were systematically examined by the research team to confirm, refute, or refine the theory using previously reported strategies such as juxtaposition, reconciliation, adjudication, and consolidation [37, 38]. Patients and Carers (public contributors) were consulted on the analysis and informed the development of the programme theory. The group was made up of 15 people, aged up to 65 years, and caring experience of between 1 and 20 years. These contributors are part of regional and local groups that provide various levels of contribution to research. For this research, public contributors helped to support the research team’s topical knowledge, CMOC development and refinement.

## Results

**Fig. 1:**
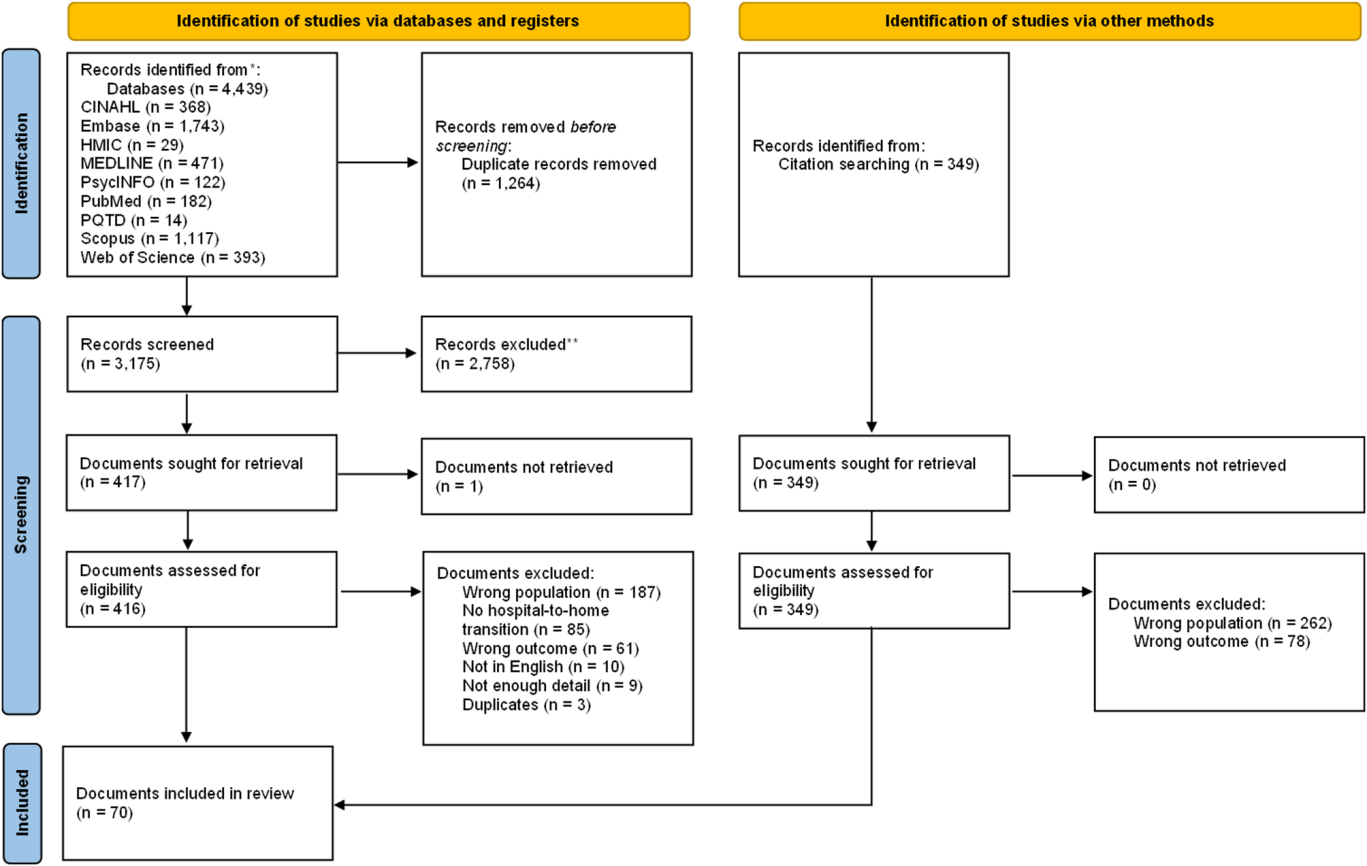
Preferred Reporting Items for Systematic Reviews and Meta-Analyses (PRISMA) Diagram showing number of included documents

Figure 1 shows the document screening process. The main search returned 3175 documents, of which 62 were included, and an additional 8 from citation searches. Documents were published between 2004 and 2024, with 49% (*n* = 34) since 2020. Most were from the USA (*n* = 32), UK (*n* = 10), Canada (*n* = 8), and Australia (*n* = 7), with remaining documents from Europe (*n* = 8) or South East Asia (*n* = 5). The most common research design explored statistical associations between MLTCiD and hospital readmission using administrative data (*n* = 28). Remaining documents included qualitative designs (*n* = 11), case studies (*n* = 10), primary quantitative designs (*n* = 6), mixed-methods (*n* = 6), commentaries (*n* = 4), systematic review (*n* = 3), scoping review (*n* = 1) and protocol (*n* = 1). Supplementary materials contain additional details of document characteristics.

### Refined programme theory

**Fig. 2:**
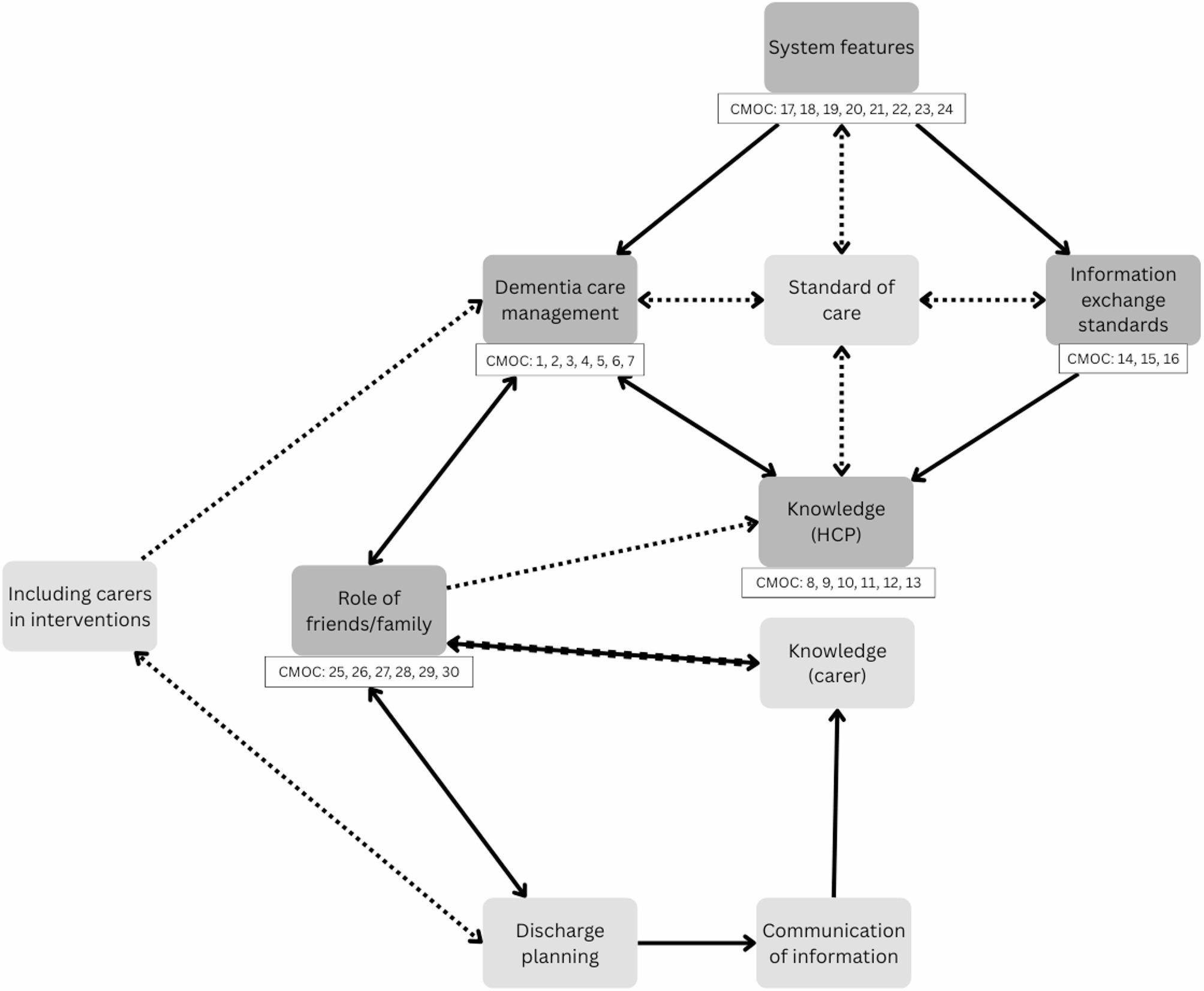
Refined programme theory. Broken line: Not enough data to support a plausible link; Unbroken line: Evidence to support plausible link; Bold line: established relationship

From synthesis of the included documents, we identified features of routine care that were integral to how hospital-to-home transitions worked. These routine features were interlinked with five features that appeared specific to MLTCiD: dementia care management, knowledge (HCP), information exchange standards, system features and role of friends/family, from which 30 CMOCs were developed (supplementary material). An explanation of these components and the corresponding CMOCs are presented below. Routine features, including carer knowledge, communication of information, discharge planning, including carers in interventions, and standard of care were not influenced by MLTCiD. The interactions between routine and dementia-specific features explain the contexts and mechanisms influencing how hospital-to-home transitions work in this population and have been represented in the refined programme theory (Fig. 2).

**Fig. 3:**
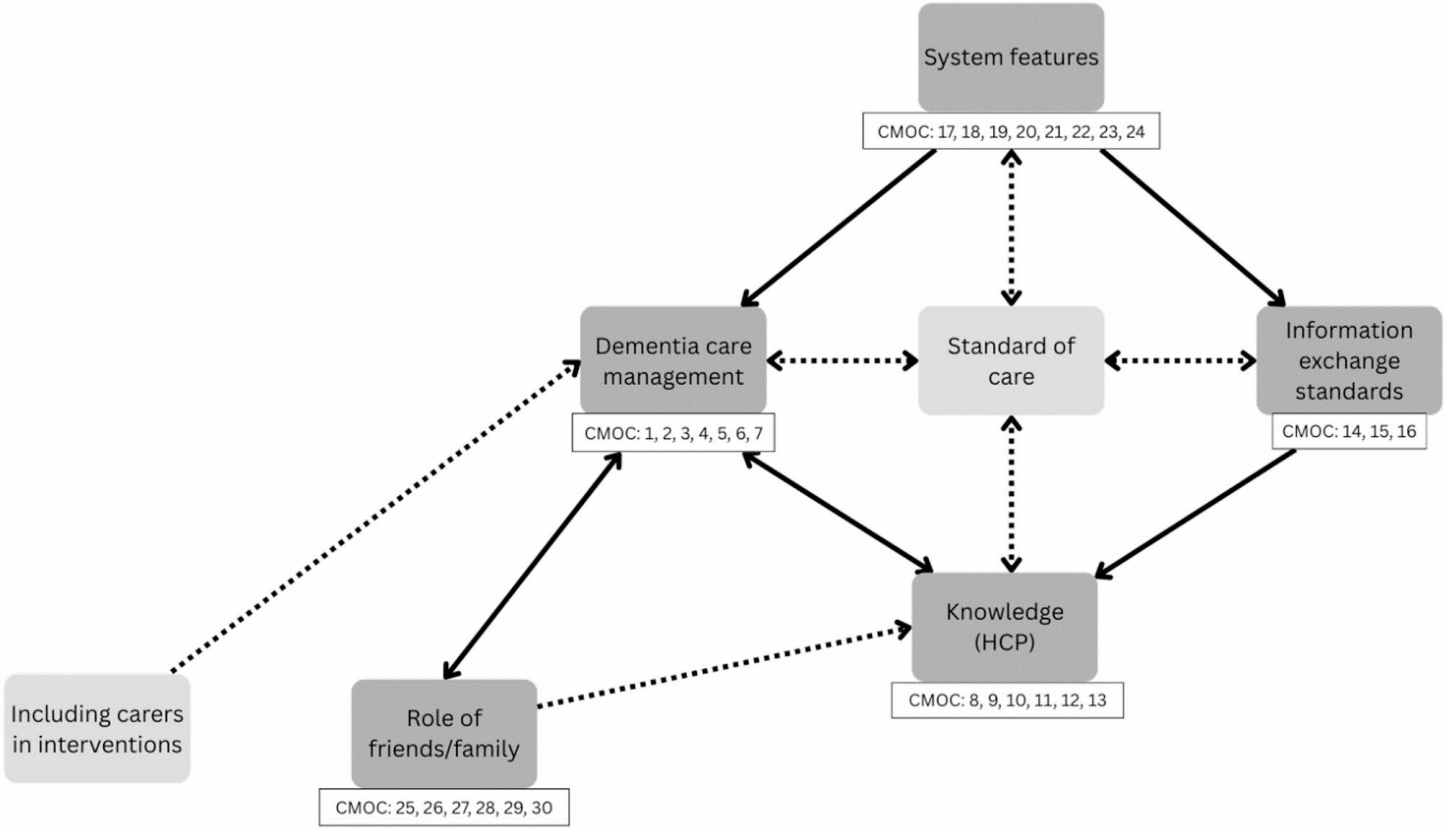
Programme theory features specific to MLTCiD. Broken line: Not enough data to support a plausible link; Unbroken line: Evidence to support plausible link

We sought to identify specific features of how hospital-to-home transitions work (or not) in relation to MLTCiD, therefore the focus of the results reflects key findings that were specific to MLTCiD rather than seeking to discuss the routine features that were also identified. Figure 3 (below) shows the programme theory components that were specific to MLTCiD. Health and social care system features (e.g. design, fragmented pathways) were associated with challenges patients faced accessing care to support their needs and influenced the support HCPs gave (CMOC 17–24). Many systems lacked a standardised approach to sharing dementia diagnoses (information exchange standards; CMOC 14–16), which influenced the knowledge HCPs had about dementia (CMOC 8–13). Dementia care management encompassed the additional care needs associated with having dementia alongside other long-term conditions, which was influenced by system-level features such as the availability of suitable resources (CMOC 1–7). Inconsistencies in the standard of care from admission and during the hospital stay (potentially explained in part by a lack of standardised information exchange) were associated with reduced patient wellbeing and potential inequitable access to care, which increased the risk of readmission [28, 39–43]. From the evidence, the standard of care patients received appeared linked to HCP knowledge of dementia broadly as well relating to the patient’s diagnosis, and dementia care management. Patients and carers reported frequent transitions between HCPs and providers as a normal part of care [28, 43, 44], with limited evidence to suggest this was part of the additional care needs associated with MLTCiD (dementia care management.

Although limited, some evidence suggested that friends/family acting as carers contributed to the knowledge HCPs had of the patient and their condition. This influenced dementia care management as HCPs with understanding of dementia were able to support carers needs and reduce stress (CMOC 1–7). This link was bidirectional, as understanding the additional needs of patients with MLTCiD influenced HCPs’ knowledge of dementia as a condition. Dementia care management was further linked to the role of friends/family, who faced increased burden associated with additional caring responsibilities. This influenced how involved friends/family were with the patient’s post-discharge care (CMOC 25–30). This link was also bidirectional, as the role friends/family took in the patient’s care influenced decisions made for patients to receive suitable treatments and to organise community support for their additional needs, highlighting a potential feedback loop.

**Fig. 4:**
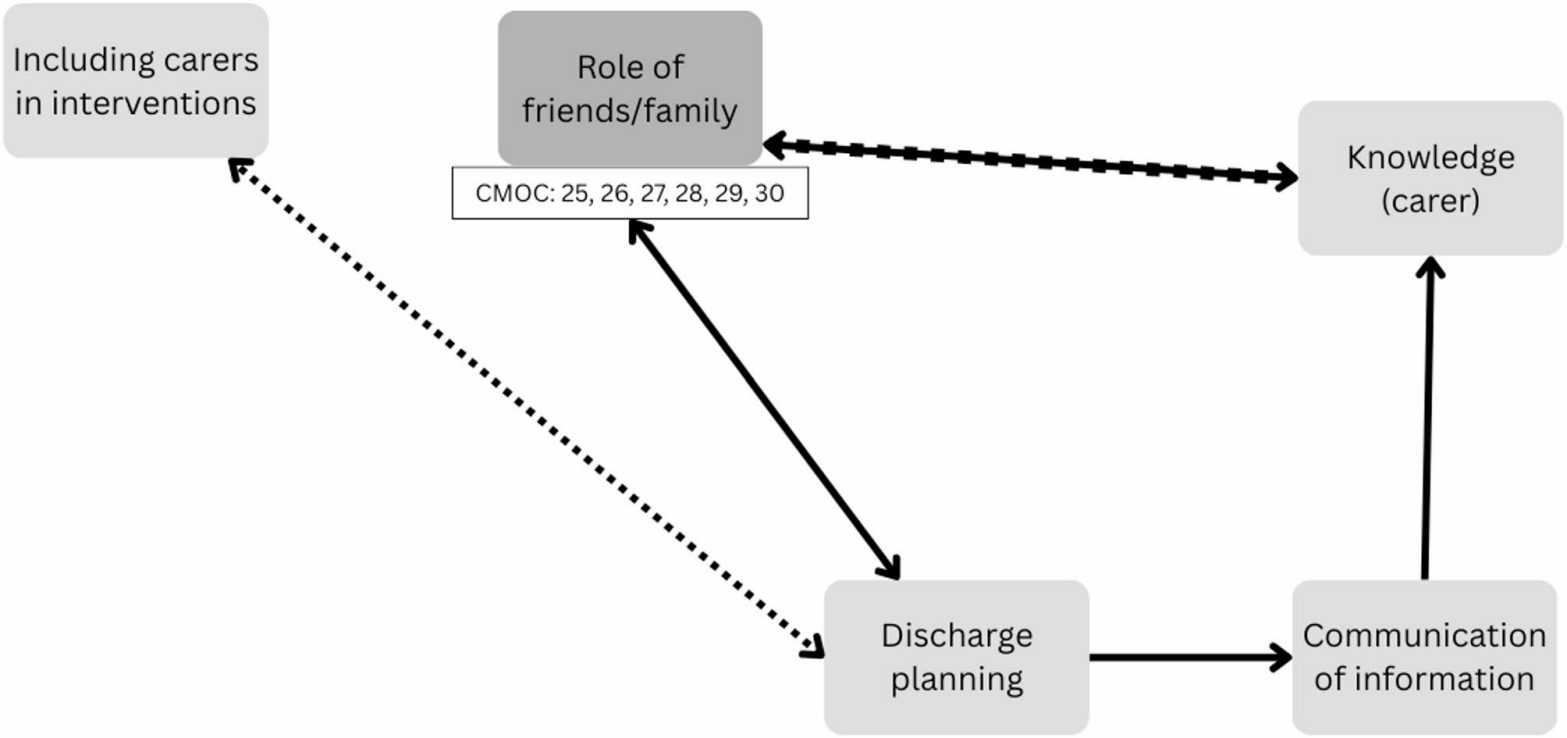
Programme theory feature of routine care. Broken line: Not enough data to support a plausible link; Unbroken line: Evidence to support plausible link; Bold line: established relationship

The second part of the programme theory represents relationships between routine features of transitions (Fig. 4). Key features of discharge planning included developing meaningful care plans, which were communicated to all involved in the transition, informed by the patient’s goals of care and provided opportunities for follow-up in the community [28, 39, 43–61]. Communication with patients and carers was facilitated by the delivery of clear, concise, and relevant information, for carers to digest in their own time. Post-discharge follow-up was linked to carers understanding of condition management, and confidence to support the patient’s needs [28, 44, 53–55, 58–60, 62–65]. Carers’ knowledge (e.g., health literacy, the patient’s care needs) was linked to their experience of carer burden, and use of urgent care after returning home, and contributed to the ways they engaged with information provided at discharge [43, 44, 57, 59, 60, 64, 66, 67]. Similarly, transitional care interventions focused on including carers before discharge to support their engagement with care at home [44, 52, 68]. Evidence to support the influence of including carers in interventions in discharge planning was limited, however this was likely to improve engagement. To engage friends/family acting in the role of carers in discharge planning, the information that is communicated to them must be accessible, and easily understood.

### Dementia care management

Supporting care management during hospital-to-home transitions was associated with increased carer burden and emotional distress, as carers felt responsible for more of the patient’s care when they had MLTCiD. Substantial cognitive decline, behavioural and psychological symptoms of dementia (BPSD) increased reliance on carers to manage care needs [28, 46, 69]. Increasing care needs due to other conditions was linked to exacerbation of dementia symptoms, increasing reliance on others, and subsequently placing more responsibility on carers (CMOC1) [28, 44, 46–48, 54, 59, 69–74]. Conversely, evidence suggested that adherence to self-management in the community was improved when patients managed other conditions before dementia onset, as cognitive decline impacted condition knowledge less (CMOC2) [27, 53]. However, dementia progression influenced the perceived appropriateness of treatments, as changing understanding of risk and benefit to the patient’s quality of life was linked to decisions not to treat certain conditions (CMOC3) [45, 60, 74].

Due to disease progression, patients’ perceptions of their own capability and condition management differed to their carers’ [45, 75]. Visual or hearing impairments further complicated communication between patients and HCPs, highlighting the need to include carers in decision-making during transitions [76]. We found evidence of HCPs supporting carers to advocate for patients by guiding them to ask relevant questions or providing medication information [52, 54]. This enabled information sharing between carers and HCPs, and improved understanding of patient needs, subsequently impacting their ability to tailor discharge planning (CMOC4) [43, 46, 52, 54, 75, 76]. Without advocates, patients with communication difficulties were often overlooked in busy hospital wards, increasing their risk of harm [54, 77]. For example, evidence linked this to poorer functional and nutritional status, and increased risk of undetected illnesses at discharge (CMOC5) [54, 77, 78].

Providing tailored and concise education about dementia care and social needs to patients and carers improved transitions by increasing understanding of post-discharge care requirements such as advance care planning [28, 44, 58, 60]. This education, which also included information on available community services, reduced carer burden due to greater confidence in their ability (CMOC6) [28, 44, 60]. However, difficulties accessing community support (e.g., disease support groups, day centres) or services that could not accommodate complex needs (e.g. hearing impairments) reduced engagement from patients and carers (CMOC7) [46].

### Knowledge (Healthcare professionals)

HCP knowledge of dementia and its interaction with the management of other conditions influenced the extent to which hospital-to-home transitions worked for older adults with MLTCiD. Many HCPs, including geriatric specialists, lacked adequate dementia training [45, 50, 53, 58, 60, 72, 73, 77, 79–82]. This reduced confidence in their ability to identify and diagnose dementia, subsequently limiting the extent to which discharge planning reflected the patient’s care needs (CMOC8) [49, 50, 58, 79–82]. This was linked to inadequate cognitive screening, poor documentation, and hospital readmission [49, 58, 79–82]. When dementia was diagnosed, limited knowledge often prevented HCPs from effectively planning care or addressing the impact of dementia symptoms on condition management (CMOC9) [46, 51, 54, 73, 74, 77, 83–85]. For example, while palliative care can support quality of life and prevent unnecessary transitions, older adults with MLTCiD were rarely referred to these services early in their dementia journey due to a lack of awareness of the benefits for patients with dementia (CMOC10) [59, 64, 85, 86]. In contrast, we found evidence of HCPs tailoring care for patients with MLTCiD by adapting diabetic medications management, based on patients’ ability to follow dosage instructions, but this approach was not widespread (CMOC11) [53, 87].

The extent of HCPs’ knowledge about MLTCiD influenced their perception of patients. When HCPs assumed patients with MLTCiD were unable to participate in rehabilitation or educational programmes, referrals were reduced, which limited access to supportive services despite evidence of potential benefits (CMOC12) [49, 53, 88, 89]. In emergency departments, HCPs frequently believed patients with MLTCiD would need social support, and did not have any urgent medical needs [77, 90]. Consequently, underlying medical complaints masked by symptoms of confusion or BPSD had potential to be overlooked (known as under-triage), increasing risk of harm and readmission [56, 60, 76, 77]. Analysis suggested that pressure to comply with the system-level focus on occupancy targets and minimising length of stay further fuelled these beliefs, as older adults with dementia typically stay longer in hospital [41, 43, 48, 50, 75, 78, 84, 89, 91–93]. Additionally, fragmentation between health and social care contributed to reduced responsibility for HCPs in emergency departments to support social and non-urgent needs, likely increasing the patient’s risk of under-triage (CMOC13) [47, 49, 56, 60, 76, 77, 82, 90, 93, 94].

### Information exchange standards (Healthcare professionals)

Care for patients with MLTCiD is addressed by numerous providers making it necessary to transfer information about the patient’s dementia diagnosis between HCPs. However, we found no standardised approach to documenting diagnosis information. Literature included the use of non-standardised terminology, (e.g., ‘confusion’, ‘cognitive impairment’) or non-compliance with labelling medical records to indicate dementia [45, 50, 79, 89]. Use of different electronic health record systems between providers presented an additional communication barrier, further limiting the transfer of diagnosis information [45, 50, 53, 66]. As a result, HCPs working outside of geriatric care were often unaware of dementia diagnoses (CMOC14) [50, 53, 59, 66, 79, 86, 89]. Patient-held records had potential to improve information sharing, but this was not standardised practice supported by healthcare systems; often patients, carers, or HCPs were unaware of such documents, resulting in non-use [53]. This suggests that without prioritisation, patient-held records are less likely to be developed to improve communication because their value is not clear to HCPs (CMOC15) [53].

Discharge education for patients with MLTCiD often targeted carers due to patients’ reduced ability to process follow-up care information, but this was rarely documented accurately [72]. As a result, HCPs in other areas were likely to assume education had been provided to the patient, leaving carers without necessary information. Standardised reporting of who had received the discharge education was advocated for, but not reported elsewhere (CMOC16) [72].

### System features

Patients with MLTCiD experienced challenges accessing care, particularly in distressing environments like emergency departments, which exacerbated BPSD [54, 72, 77]. Carers found supporting patients during long waits with limited HCP intervention difficult, increasing carer burden (CMOC17) [54, 77]. Elevated BPSD often masks the patient’s care needs, leading to misrecognition and inaccuracies in the care provided by HCPs (CMOC18) [45, 72, 77, 93].

Post-discharge support was often required from multiple providers, which did not align with single-condition care pathways. As a result, collaboration between HCPs was associated with delayed discharges, emphasising the problem in care co-ordination (CMOC19) [40, 42, 74, 83, 88, 91, 92]. In several instances HCPs were unable to co-ordinate support from community healthcare services (e.g. district nursing) due to separation between departments [43, 45, 46]. Fragmentation between health and social care further contributed to unmet needs, as the distribution of responsibilities limited the care needs covered by support packages from providers (e.g. personal care), creating difficulties organising post-discharge care [45, 90]. Furthermore, support was often short-term, leaving service gaps for those with long-term care needs [46, 53, 80]. This was rarely inclusive of dementia-specific needs, such as reducing the number of different visitors at home or fitting around the patient’s routine to reduce distress, which was linked to increased readmissions [43, 54, 95, 96]. In some cases, HCPs developed ‘workarounds’ to address service gaps, such as discharging and readmitting patients into support programmes, but this was not a system-based approach [43, 53, 80]. When HCPs did not develop ‘workarounds’, patients were discharged into unsafe living situations, and readmissions were increased [90, 97] (CMOC20).

There was evidence to suggest that those with MLTCiD use more unpaid care than those with dementia or LTCs alone, which has increased over time [53]. This suggests that, in the absence of inclusive pathways, friends and family took on greater responsibilities to support the patient (CMOC21) [53]. Patients without carers experienced challenges during transitions, as arranging transport or follow-up care was often not considered HCPs’ responsibility, creating a service gap (CMOC22) [27, 45, 46, 53]. Confusion over roles between health and social care professionals further exacerbated this issue.

Some systems aimed to improve patient safety during transitions by assigning HCPs to facilitate care coordination. This included acting as a single point of contact in the initial post-discharge period, to improve communication, reduce confusion and subsequently prevent avoidable rehospitalisation, however evidence of their effectiveness was limited (CMOC23) [53, 55, 60]. Documents advocated for a holistic approach centred on dementia [60, 65, 76, 77, 98]. For example, integrated memory care combining condition reviews with symptom management showed promise in reducing readmissions and urgent care use, which were not affected by number of conditions [65, 98]. This emphasised a need to organise care around dementia to support transitions, as improved HCP understanding of the patient’s care needs facilitated condition management (CMOC24) [44, 60, 76, 77, 98].

### Role of friends/family

Friends and family were integral to each theme, often acting as unpaid carers for individuals with MLTCiD and playing a central role in discharge planning and information sharing. Feelings of obligation influenced acceptance of the carer role; cultural norms, in which family members typically look after their elder relatives, likely contributed to this, as expectations were stronger for closer relatives (e.g. children) to assume responsibility for care (CMOC25) [40, 46]. Expectations were highlighted when patients refused paid carer support (e.g. formal carers) after transitioning home, resulting in children of the patient taking additional caring responsibilities (CMOC26) [44]. Carers often supported patients by relaying information and monitoring for changes in symptoms, however in several instances HCPs also assumed that they would take up medical care responsibilities after discharge, such as administering treatments, monitoring blood sugar, and giving insulin injections [44, 46, 47, 53]. Carers often received no training for these skills, and were frequently reported as unhappy, uncomfortable, and unprepared to deliver this support [44, 46, 47, 53]. Subsequently, this was linked to emotional distress in carers, and increased hospital readmissions for patients when care needs were unmet (CMOC27) [44, 46, 47, 53, 67].

When multiple carers were involved, differences of opinion created uncertainty and hindered decision-making on the patient’s behalf (CMOC28) [46, 99]. This was further complicated when patients were unable to communicate their needs, as fear of missing symptoms and uncertainty had potential to inhibit care management at home, and contributed to carer burden (CMOC29) [27]. Interventions to support carers were limited, though online self-care education showed potential to improve mental health, wellbeing, and self-efficacy, helping carers feel more confident in their roles, and supported higher engagement in patient-focused interventions (CMOC30) [28, 44, 52, 63, 68, 100, 101].

## Discussion

### Summary of findings

This review aimed to develop a theory explaining how, for whom, and to what extent hospital-to-home transitions work for older adults with MLTCiD. Our findings highlighted features that were integral to how transitions should work, with a subset of five components specific to MLTCiD (dementia care management, knowledge, information exchange standards, system, and the role of friends/family) providing important contexts influencing their success. Carers faced increased burden and emotional distress as MLTCiD was associated with greater responsibilities during transitions, particularly when patients had BPSD or increasing care needs due to other conditions. Limited dementia training for HCPs undermined their ability to provide appropriate discharge planning and effectively manage care needs. Lack of standardised documentation and communication of dementia diagnoses led to inadequate transfers of information, hindering care continuity and effective discharge planning. Fragmented care pathways and inadequate collaboration between providers resulted in delays, unsafe discharges, and increased reliance on carers, exacerbating service gaps for patients with MLTCiD. Carers often felt obligated to take on medical and caregiving responsibilities without adequate training, contributing to stress, decision-making conflicts, and potential hospital readmissions.

### Comparison with literature

The Transitional Care Model (TCM) is the predominant theory providing a framework to improve outcomes for older adults with complex care needs as they move between healthcare settings [10]. This model supports transitions for older adults, however findings from this review add greater detail on how these processes work with MLTCiD. For example, the model advocates for comprehensive, holistic assessment of the patient’s needs, which is widely supported in the literature [60, 65, 76, 77, 98]. Our findings suggest that in practice however, admission and discharge procedures prioritise the admission diagnosis, and do not adequately account for dementia symptoms (e.g. cognitive decline, BPSD) [47, 49, 56, 60, 76, 77, 90, 93]. As a result, care plans frequently fail to integrate dementia with the management of co-existing conditions, increasing the risk of fragmented care and unmet needs. This has been reflected in the wider research, which suggests that hospital discharge procedures do not support the higher levels of coordination, communication and consultation required in complex discharge planning, leaving service gaps for those with dementia [102]. Tailored interventions for older adults with MLTCiD were rarely implemented in the reviewed documents, despite their advantages in reducing hospital readmissions and carer burden [28, 44, 60]. In the wider literature, most transitional care interventions have not focused on dementia [103]; indeed, patients with dementia or complex care needs are often excluded from transition research [104]. Furthermore, when interventions exist, they primarily focus on outcomes such as healthcare resource use or cost rather than elements of care coordination [105].

Continuity of care is central to the success of hospital-to-home transitions. An essential element of the TCM are trained HCPs, who function as care coordinators to oversee transitions and ensure continuity of services post-discharge [10]. Our findings suggest that there is a clear lack of appropriate knowledge and training for HCPs on dementia, which had a knock-on effect on discharge planning and patient access to services [46, 50, 51, 54, 59, 61, 73, 74, 77, 78, 80–86]. However, high demand on healthcare services combined with approaches to care that focus on physical needs and meeting compliance targets is thought to have created a disease-orientated culture of care in which dementia is not a priority [106]. Research has also found that HCPs responsible for discharge planning are more likely to learn ‘on the job’ and training programmes often lack follow-up over time, limiting sustained improvements [102, 107, 108]. Inconsistent communication further contributed to gaps in continuity. Our review highlighted the lack of a standardised approach to recording and reporting dementia diagnoses during hospital-to-home transitions, which was fuelled by fragmentation and lack of interfacing between disparate electronic health record systems. In this review, assigning a single care coordinator was suggested as a potential solution, but rarely implemented [53, 55, 60, 99]. Whilst care coordination through a single point of contact has been a longstanding recommendation, evidence is limited to support their use [106, 109]. In contrast to the model’s recommendation and our findings, some research suggests that dementia care coordination is mostly a non-clinical role placed in the community, who are likely to face barriers due to access, and lack of standardised documentation [110, 111].

The TCM recommends active engagement of carers through communication and involvement in setting care goals, offering education and resources to empower them during transitions [10]. Carers are recognised as important and often face increased burden due to the additional challenge of managing MLTCiD. However, we found that many carers felt unsupported and unprepared, especially when required to take on medical responsibilities without formal training. Existing structures that had been adapted for dementia (e.g., online education programmes) reported some improvement in preparing carers to support the patient’s needs during transitions, but often neglected to provide health and wellbeing support for the carer [28, 44, 52, 63, 68, 100, 101]. In the literature, transitional care support remains patient-focused, which may mean that interventions to improve capability to care may be limited by a failure to support the needs of the carer [112]. Despite several reports that carers were uncomfortable, or did not want to support additional needs after discharge, only one of the reviewed documents advocated for care pathways that supported carers to have choice in providing post-discharge care [53]. We found some evidence of HCPs supporting carers to advocate for the patient’s needs, which led to developing more accurate goals of care. In contrast, when patients had no carer involvement our findings suggested they would be less able to navigate transitions. We found few documents had investigated transitions for those without support. Living alone with dementia without carer involvement has been associated with worse health outcomes, such as lower life satisfaction and delayed access to services [113]; as those without carers are often excluded from research this highlights a gap in our understanding of how to assist those returning home without a network to support their needs [113].

### Implications

Fragmentation between health and social care creates barriers to successful hospital-to-home transitions for patients, carers, and HCPs. We found that poor communication and unclear division of responsibilities between HCPs and social care often left patients with MLTCiD without adequate discharge planning and follow-up care, highlighting a need for system-level integration. Allocating responsibility for this through a single point of contact (such as a coordinator) may improve communication and continuity of care and reduce confusion for patients and carers. Standardised reporting of patient information, which may be enabled by digital technologies, is necessary for successful transitions [114]. Dementia-specific training should be provided to HCPs responsible for discharge planning and in areas of frequent attendance (e.g. urgent care) to improve the accuracy of post-discharge support. Addressing the skills gap in dementia knowledge and training has been a longstanding policy initiative in the UK [107]. However, without system-level changes to the culture of care and prioritisation of patients with dementia, it is unlikely that education programmes alone will make a difference to the success of hospital-to-home transitions. Decision-making support for HCPs, that is person-centred, able to improve understanding of the patient’s care needs and challenge existing beliefs about dementia, is required. We found little evidence of transitional care interventions that had been tailored to MLTCiD. Research investigating dementia-specific hospital-to-home interventions is in an early stage, and as older adults with MLTCiD have an increased risk of harm during transitions, their inclusion in interventions is important to understand how we can support their needs [103, 104]. This is particularly relevant for older adults with MLTCiD without supportive networks. Finally, we found hospital-to-home interventions often neglected the health and wellbeing needs of carers, which may limit their capability to support the patient. Considering the additional responsibilities associated with MLTCiD, support for carers’ wellbeing and welfare should be incorporated into future transitional care interventions.

### Strengths and limitations

This is the first review to synthesise and develop theories about hospital-to-home transitions for adults with MLTCiD, and those involved in their care. Due to the limited amount of published research in this area, a realist review enabled the capture of relevant information from a multitude of sources that would not be included in other traditional reviews. The majority of documents included in this review came from the USA (*n* = 32) or other English-speaking countries (*n* = 25), which may limit the extent to which the findings can be generalised between health and social care systems that work differently worldwide. This further highlights the need for additional research in countries with less-developed dementia services. CMOC development was strengthened with input from patients, carers, and HCPs with experience of transitions. A further strength of realist approaches is their ability to account for complexity, which is relevant and appropriate to understanding hospital-to-home transitions in this population with complex needs [31]. However, due to this complexity, the final programme theory can only represent the available knowledge, which may present a partial understanding. Additionally, only articles published in the English language were considered for inclusion, which may have excluded relevant research. We identified two grey literature documents from a systematic search of two large grey literature databases. Additional documents may have been retrieved from reviewing websites which is a limitation of this research and could be more purposively included if further review and refinement of the programme theory is desired. Palliative care was not the focus of this review, which may have revealed important insights to understanding how this population experience transitions of care. Future research is needed to explore this in depth. This review is the first part in a series of work that intends to understand what works and does not work for this population in hospital-to-home transitions and identify how best to support those involved. In comparison to previous research that identified key elements influencing transitions from synthesis of quantitative data, this review provides an explanation of the context surrounding these factors among others, to improve understanding of why this population experiences specific outcomes during hospital-to-home transitions [28]. This detailed understanding of the contexts under which these transitions occur which will be supported by additional research to develop suitable, supportive guidance.

## Conclusion

Hospital-to-home transitions are a complex issue for older adults with MLTCiD and those involved in their care with no simple solution. However, the use of a realist approach has allowed us to recognise this complexity in order to understand the specific contexts involved for this population. Fragmentation between health and social care, poor communication, and a lack of dementia-specific training for HCPs were key contexts associated with inadequate discharge planning, follow-up care, and increased risk of harm for patients, especially those without supportive networks. System-level integration, including standardised reporting is essential to addressing these gaps. Tailored interventions for patients with MLTCiD remain underdeveloped, highlighting an urgent need for research into dementia-specific approaches. Future transitional care interventions must address needs of patients with MLTCiD and the health and wellbeing of their carers to improve outcomes and reduce the risks associated with hospital-to-home transitions.

## Electronic supplementary material

Below is the link to the electronic supplementary material.

Supplementary Material 1

## Data Availability

No datasets were generated or analysed during the current study.

## Abbreviations

CMOC: context-mechanism-outcome configuration
HCP: Healthcare professional
IPT: Initial programme theory
MLTC: Multiple long-term conditions
MLTCiD: Multiple long-term conditions including dementia
NICE: National Institute for Health and Care Excellence
NIHR: National Institute for Health and Care Research
UK: United Kingdom
