# Supplementary material for: Do hospital-to-home transitions work for older adults with multiple long-term conditions including dementia? A realist review
Source: BMC Geriatr. 2025 Jul 9;25:511. doi: 10.1186/s12877-025-06123-0 (PMC12239264; doi:10.1186/s12877-025-06123-0)
Supplement: Supplementary file 1 — Supplementary Material 1 [file 12877_2025_6123_MOESM1_ESM.docx]

Do Hospital-to-Home Transitions Work for Older Adults with Multiple Long-Term Conditions Including Dementia? A Realist Review

Supplementary Materials

Contents

[Appendix 1. PRISMA 2020 checklist 3](#_Toc188866798)

[Appendix 2. Search strategy 6](#_Toc188866799)

[Appendix 3. Characteristics table 7](#_Toc188866800)

[Appendix 4. CMOCs and illustrative data 23](#_Toc188866801)

[Appendix 5. Supplementary reference list 30](#_Toc188866802)

# Appendix 1. PRISMA 2020 checklist

| **Section and Topic** | **Item #** | **Checklist item** | **Reported on page #** |
| --- | --- | --- | --- |
| **TITLE** | | |  |
| Title | 1 | Identify the report as a systematic review. | 1 |
| **ABSTRACT** | | |  |
| Abstract | 2 | See the PRISMA 2020 for Abstracts checklist. | 2 |
| **INTRODUCTION** | | |  |
| Rationale | 3 | Describe the rationale for the review in the context of existing knowledge. | 4, 5 |
| Objectives | 4 | Provide an explicit statement of the objective(s) or question(s) the review addresses. | 5 |
| **METHODS** | | |  |
| Eligibility criteria | 5 | Specify the inclusion and exclusion criteria for the review and how studies were grouped for the syntheses. | 7,8 |
| Information sources | 6 | Specify all databases, registers, websites, organisations, reference lists and other sources searched or consulted to identify studies. Specify the date when each source was last searched or consulted. | 7 |
| Search strategy | 7 | Present the full search strategies for all databases, registers and websites, including any filters and limits used. | 7, Supp |
| Selection process | 8 | Specify the methods used to decide whether a study met the inclusion criteria of the review, including how many reviewers screened each record and each report retrieved, whether they worked independently, and if applicable, details of automation tools used in the process. | 7,8 |
| Data collection process | 9 | Specify the methods used to collect data from reports, including how many reviewers collected data from each report, whether they worked independently, any processes for obtaining or confirming data from study investigators, and if applicable, details of automation tools used in the process. | 7, 8 |
| Data items | 10a | List and define all outcomes for which data were sought. Specify whether all results that were compatible with each outcome domain in each study were sought (e.g. for all measures, time points, analyses), and if not, the methods used to decide which results to collect. | 7, 8 |
|  | 10b | List and define all other variables for which data were sought (e.g. participant and intervention characteristics, funding sources). Describe any assumptions made about any missing or unclear information. | 7, 8 |
| Study risk of bias assessment | 11 | Specify the methods used to assess risk of bias in the included studies, including details of the tool(s) used, how many reviewers assessed each study and whether they worked independently, and if applicable, details of automation tools used in the process. | 7, 8 |
| Effect measures | 12 | Specify for each outcome the effect measure(s) (e.g. risk ratio, mean difference) used in the synthesis or presentation of results. | N/A |
| Synthesis methods | 13a | Describe the processes used to decide which studies were eligible for each synthesis (e.g. tabulating the study intervention characteristics and comparing against the planned groups for each synthesis (item #5)). | N/A |
|  | 13b | Describe any methods required to prepare the data for presentation or synthesis, such as handling of missing summary statistics, or data conversions. | N/A |
|  | 13c | Describe any methods used to tabulate or visually display results of individual studies and syntheses. | 8 |
|  | 13d | Describe any methods used to synthesize results and provide a rationale for the choice(s). If meta-analysis was performed, describe the model(s), method(s) to identify the presence and extent of statistical heterogeneity, and software package(s) used. | 7, 8 |
|  | 13e | Describe any methods used to explore possible causes of heterogeneity among study results (e.g. subgroup analysis, meta-regression). | N/A |
|  | 13f | Describe any sensitivity analyses conducted to assess robustness of the synthesized results. | N/A |
| Reporting bias assessment | 14 | Describe any methods used to assess risk of bias due to missing results in a synthesis (arising from reporting biases). | N/A |
| Certainty assessment | 15 | Describe any methods used to assess certainty (or confidence) in the body of evidence for an outcome. | 7, 8 |
| **RESULTS** | | |  |
| Study selection | 16a | Describe the results of the search and selection process, from the number of records identified in the search to the number of studies included in the review, ideally using a flow diagram. | 8, 9 |
|  | 16b | Cite studies that might appear to meet the inclusion criteria, but which were excluded, and explain why they were excluded. | 9 |
| Study characteristics | 17 | Cite each included study and present its characteristics. | 8, 9, Supp |
| Risk of bias in studies | 18 | Present assessments of risk of bias for each included study. | N/A |
| Results of individual studies | 19 | For all outcomes, present, for each study: (a) summary statistics for each group (where appropriate) and (b) an effect estimate and its precision (e.g. confidence/credible interval), ideally using structured tables or plots. | N/A |
| Results of syntheses | 20a | For each synthesis, briefly summarise the characteristics and risk of bias among contributing studies. | N/A |
|  | 20b | Present results of all statistical syntheses conducted. If meta-analysis was done, present for each the summary estimate and its precision (e.g. confidence/credible interval) and measures of statistical heterogeneity. If comparing groups, describe the direction of the effect. | N/A |
|  | 20c | Present results of all investigations of possible causes of heterogeneity among study results. | N/A |
|  | 20d | Present results of all sensitivity analyses conducted to assess the robustness of the synthesized results. | N/A |
| Reporting biases | 21 | Present assessments of risk of bias due to missing results (arising from reporting biases) for each synthesis assessed. | N/A |
| Certainty of evidence | 22 | Present assessments of certainty (or confidence) in the body of evidence for each outcome assessed. | N/A |
| **DISCUSSION** | | |  |
| Discussion | 23a | Provide a general interpretation of the results in the context of other evidence. | 19-22 |
|  | 23b | Discuss any limitations of the evidence included in the review. | 23 |
|  | 23c | Discuss any limitations of the review processes used. | 23 |
|  | 23d | Discuss implications of the results for practice, policy, and future research. | 22 |
| **OTHER INFORMATION** | | |  |
| Registration and protocol | 24a | Provide registration information for the review, including register name and registration number, or state that the review was not registered. | 6 |
|  | 24b | Indicate where the review protocol can be accessed, or state that a protocol was not prepared. | 6 |
|  | 24c | Describe and explain any amendments to information provided at registration or in the protocol. | N/A |
| Support | 25 | Describe sources of financial or non-financial support for the review, and the role of the funders or sponsors in the review. | 25 |
| Competing interests | 26 | Declare any competing interests of review authors. | 26 |
| Availability of data, code and other materials | 27 | Report which of the following are publicly available and where they can be found: template data collection forms; data extracted from included studies; data used for all analyses; analytic code; any other materials used in the review. | 7, 8 |

*From:*  Page MJ, McKenzie JE, Bossuyt PM, Boutron I, Hoffmann TC, Mulrow CD, et al. The PRISMA 2020 statement: an updated guideline for reporting systematic reviews. BMJ 2021;372:n71. doi: 10.1136/bmj.n71. This work is licensed under CC BY 4.0. To view a copy of this license, visit <https://creativecommons.org/licenses/by/4.0/>

# Appendix 2. Search strategy

MEDLINE

1. exp Dementia/ or exp AIDS Dementia Complex/ or exp Frontotemporal Dementia/ or exp Dementia, Vascular/
2. exp Chronic Disease/ or exp Multimorbidity/ or exp Comorbidity/ or multimorbid*.mp.
3. ("long term condition" or "long term conditions" or multicomorbid* or polymorbid* or "patient complexity" or syndemic or "multiple chronic condition" or "multiple chronic conditions").mp. [mp=title, book title, abstract, original title, name of substance word, subject heading word, floating sub-heading word, keyword heading word, organism supplementary concept word, protocol supplementary concept word, rare disease supplementary concept word, unique identifier, synonyms, population supplementary concept word, anatomy supplementary concept word.
4. exp Diabetes Mellitus, Type 2/
5. exp Hypertension/
6. exp Heart Failure/ or chronic heart failure.mp.
7. exp Cardiovascular Diseases/
8. exp Stroke/
9. exp Parkinson Disease/
10. exp Epilepsy/
11. exp Depression/
12. exp Asthma/
13. exp Pulmonary Disease, Chronic Obstructive/
14. exp Osteoarthritis/
15. exp Osteoporosis/
16. exp Thyroid Diseases/
17. exp hospital to home transition/ or exp patient discharge/ or exp patient handoff/ or exp patient transfer/ or exp transitional care/
18. exp "delivery of health care, integrated"/ or health services accessibility/ or exp access to primary care/
19. "integrated care".mp.
20. transition*.mp.
21. 2 or 3 or 4 or 5 or 6 or 7 or 8 or 9 or 10 or 11 or 12 or 13 or 14 or 15 or 16
22. 17 or 18 or 19 or 20
23. 1 and 21 and 22

# Appendix 3. Characteristics table

| Authors (Year) | Topic | Population | Source | Study Design | Country | Summary |
| --- | --- | --- | --- | --- | --- | --- |
| Agarwal et al. (2016) (59) | Hospital Readmission | 241 patient encounters at discharge | Peer reviewed article, primary & secondary | Quantitative, prospective cohort & retrospective chart review using administrative data | USA | Cognitive impairment is frequently undocumented and may indicate a greater risk of readmission for individuals with heart failure. |
| Agostinho et al. (2017) (71) | Hospital Readmission | 100 patient records (median 5 conditions) | Conference Abstract, secondary | Quantitative, retrospective analysis using administrative data | Portugal | The presence of MLTC correlated significantly with post-discharge adverse events in patients admitted to hospital with HF. Dementia associated with readmission. |
| Anderson et al. (2022) (96) | Hospital Readmission | 211,698 PlwD, 45/50 common chronic conditions p<.001 in PlwD) | Peer reviewed article, secondary | Quantitative, retrospective analysis of administrative data | USA | Patients discharged to the community had an elevated risk of adverse outcomes post-discharge. Diagnosed dementia was associated with increased risk of readmission within 30 days. |
| Ashbourne et al. (2021) (44) | Transitions of care | 12 PlwD (mean 3 LTCs) 29 Carers | Peer reviewed article, primary | Qualitative, interviews | Canada | Developed a theoretical framework outlining contexts, processes and influencing factors of care transitions. |
| Ashley et al. (2021) (46) | Coordination of care | 17 PlwD & cancer 22 Carers 19 HCP | Peer reviewed article, primary | Qualitative, ethnography | UK | Overarching theme of: ‘accumulated complexity. 1) Working without the full picture; 2) Difficulty and diffusion of treatment decision-making; 3) Balancing person versus process; 4) Burden of navigating cancer care; 5) Reliance on supportive family networks. |
| Baird et al. (2019) (81) | Self-management | 12 health and social care professionals in ambulatory/dementia care | Peer reviewed article, primary | Qualitative, interview | Australia | Healthcare system was described as complex, not dementia-friendly, and not accommodating of the needs of those with MTLCiD. LTC support programmes did not undertake routine cognitive assessments or have guidelines to support those with cognitive impairment. |
| Boltz et al. (2015) (69) | Transitions of care | 84 PlwD-Carer Dyads (mean 3.7 comorbidity index) | Peer reviewed article, primary | Quantitative, RCT | USA | Family-centred, function-focused care intervention in hospitalised MLTCiD with carers showed improvements in ADLs and walking performance, reductions in severity of delirium and hospital readmission. Carers showed increased preparedness for caregiving and less anxiety but no significant differences in depression, strain and mutuality. |
| Boltz et al. (2023) (53) | Transitions of care | 455 PlwD-carer dyads (mean 4.6 comorbidity index) | Peer reviewed article, primary | Quantitative, RCT | USA | Family-centred, function-focused care intervention was associated with return to baseline functioning, fewer symptoms of distress, but no differences in the physical activity, depressive symptoms and delirium severity. The intervention reported a small increase in carers' preparedness for caregiving, but no differences in anxiety, strain and burden. |
| Bronskill et al. (2020) (92) | Health care utilisation | 62,622 PlwD, 80% 6+ LTCs | Peer reviewed article, secondary | Quantitative, retrospective analysis of administrative data | Canada | Patients had increased use of home care, emergency department use, ad hospitalisations with a discharge delay. Hospitalisations with discharge delays decreased over time. |
| Browne et al. (2024) (98) | Hospital readmission | N/A | Peer reviewed article, secondary | Systematic review | UK | Poorly defined roles and responsibilities of health and social care professionals, alongside poor communication during transitions of care increases the risk of readmission in MLTCiD. |
| Bunn et al. (2016) (54) | Care pathways | 28 PlwD +Stroke/Visual impairment/diabetes, 33 carers, 56 HCPs | Report, primary and secondary | Mixed-methods, scoping review, interviews, retrospective analysis of administrative data | UK | There is a lack of continuity in healthcare systems for MLTCiD, with little integration or communication between teams. Unpaid care use in MLTCiD increased over the last decade. Qualitative data supported review findings of poor communication and no standardised approach to information sharing. |
| Chao et al. (2020) (72) | Hospital Readmission | 74 PlwD (mean 3 LTCs) with 310 CI and 258 normal comparators | Peer reviewed article, secondary | Quantitative, retrospective analysis of administrative data | Taiwan | Number and severity of LTCs were associated with length of stay and readmission for PlwD/CI. Older hospitalized patients with cognitive disorders had worse clinical outcomes compared with those without cognitive disorders. |
| Clark et al. (2018) (63) | Integrated Dementia Care | 81F, AD + 4 LTC | Peer reviewed article, primary | Case Study | USA | Describes a case example in an integrated dementia care pathway, to reduce transitions of care and build trusting relationships between the patient, carer and HCPs. |
| Clevenger et al. (2018) (99) | Integrated Dementia Care | 139 PlwD dyads (M 5.8 LTCs) | Peer reviewed, primary | Quantitative, use data, survey | USA | Reported reduction in ED visits and ambulatory-sensitive hospitalisations, highlighting potential benefit of integrated palliative care approach to care coordination. |
| Cumbler et al. (2008) (41) | Transitions of care | 86F, dementia + 6 LTCs | Peer reviewed article, primary | Case Study | USA | Describes discharge process with no case management or support for the patient, resulting in medications not delivered post-discharge and readmission. Highlights deficits in communication as one barrier to successful hospital-to-home transition. |
| Daiello et al. (2014) (97) | Hospital Readmission | 914 PlwD (78.6% cohort <3 LTCs, 21.4% >3) | Peer reviewed, secondary | Quantitative, retrospective analysis of administrative data | USA | Hospitalised patients with dementia had significantly more diagnoses, and more common 30-day readmission after controlling for age, MLTC, medication and previous admissions. |
| Digby et al. (2018) (91) | Hospital outcomes | 29 nurses | Peer reviewed article, primary | Qualitative, interview & observation | Australia | Overall: The 'unworthy' patient, 4 themes: 1) Patients with dementia and hospital performance targets; 2) Patients with dementia and risk; 3) Patients with dementia and nurse workload; 4) Patients with dementia and acute care issues. |
| Dooley et al. (2020) (116) | Urgent Care | N/A | Peer reviewed article, secondary | Scoping Review | UK | Reported complex care scenarios influencing urgent care use. Lack of understanding of dementia and knowledge about the patient, inadequate community support, and competing demands of informal and professional carers create additional challenges to receiving appropriate care. |
| Duah-Owusu et al. (2023) (93) | Discharge Planning | 32 unpaid carers 20 hospital staff | Peer reviewed article, primary | Qualitative, interview | UK | Several factors were highlighted as having a positive or negative impact on discharge planning: patient cognitive capacity, staff communication skills, discharge meetings, and the available equipment. |
| Duggleby et al. (2017) (101) | Carer Wellbeing | N/A | Protocol | N/A | Canada | Protocol for a mixed-methods RCT using a digital tool to support carers of MLTCiD through care transitions. |
| Duggleby et al. (2018) (102) | Carer Wellbeing | 199 carers | Peer reviewed article, primary | Mixed methods, RCT, survey, interview | Canada | No significant differences between groups on mental component score, but significant differences observed on measure of carer's feelings of hope, suggesting positive influence of intervention. |
| Duggleby et al. (2019) (64) | Carer Wellbeing | 92 carers | Peer reviewed article, primary | Mixed-methods, survey, interview | Canada | Carers using the intervention reported higher self-efficacy than non-users. Reasons for non-use include caregiving demands, problems accessing the site and preferences for other formats. |
| Elliot et al. (2021) (67) | Medication management | 86F, dementia + 5 LTCs | Peer reviewed article, primary | Case Study | USA | Describes a case example of inappropriate OTC medication use, polypharmacy and communication barrier in the coordination of care during hospital-to-home transitions. |
| Fleming et al. (2013) (56) | Transitions of care | 84F, dementia + 1 LTC | Peer reviewed article, primary | Case Study | USA | Describes the impact of a care transitions coordinator on discharge planning and preventable admissions. Average readmission rates at 12-months decreased from 17% to 12% with the programme. |
| Gilmore-Bykovskyi et al. (2018) (80) | Documentation of Dementia | 343 PlwD, (comorbidity index mean 6.94) | Peer reviewed article, secondary | Quantitative, retrospective analysis of administrative data | USA | Narrative text was commonly used to describe symptoms of cognitive impairment in MLTCiD, including vague terms not consistent with standardised instruments or diagnostic criteria. General statements may reflect of inadequate training, awareness and/or assessment of other key areas of cognition. |
| Gilmore-Bykovskyi et al. (2023) (42) | Hospital Readmission | 215 Black PlwD (rehospitalisation index 40.9) | Peer reviewed article, secondary | Quantitative, retrospective analysis of administrative data | USA | Black Medicare beneficiaries had 37% higher readmission odds than white beneficiaries. Readmission risk persisted after adjustment for geographic, social, hospital, stay-level and comorbidity characteristics. |
| Graversen et al. (2021) (57) | Hospital Readmission | 25,948 PlwD (63.2% 3+ LTC) | Peer reviewed article, secondary | Quantitative, retrospective analysis of administrative data | Denmark | Dementia was associated with higher short-term mortality after pneumonia in those using anti-psychotics, and slightly higher readmission, more so in the first days after discharge. |
| Henkle et al. (2023) (117) | Self-management | 47 participants, 29 MoCA <26 + COPD | Peer reviewed article, primary | Quantitative, prospective observational | USA | CI is highly prevalent in COPD outpatients with recent exacerbations and is associated with poor inhaler technique. |
| Hesselink et al. (2012) (76) | Discharge Planning | 192 interviews, 26 focus groups with patients, family members, hospital physicians & nurses, community GP physicians & nurses | Peer reviewed article, primary | Qualitative, interview, focus groups | Multicentre (Netherlands, Spain, Poland, Italy, Sweden) | Patient and family involvement in discharge planning is determined by how willing care providers are to accommodate their capabilities, needs and preferences. Future interventions directed at HCPs attitudes and organisation leadership, are needed, to improve communication among care providers, patients and families, and between hospital and community care providers. |
| Holley et al. (2008) (65) | Coordination of care | 74 PlwD (2-4 LTCs), 22 carers | Peer reviewed article, primary | Mixed-methods, Chart review, interview | USA | Themes identified from qualitative analysis included a desire to remain at home, access to geriatric and palliative medicine specialists, and challenges during transitions of care. Amount of care required and satisfaction with the programme were generally high. |
| Horvitz et al. (2011) (86) | Hospital outcomes | 150 patients, with LTC and stroke/hip fracture/'disabling event' | Conference Abstract, primary | Quantitative, retrospective analysis of administrative data | Israel | Comorbidity should be accounted for in the prediction of walking outcomes, especially dementia and history of previous fractures. |
| Ibrahim et al. (2017) (62) | Care pathways | N/A | Peer reviewed article | Commentary | Australia | Developed framework with 5 key processes describing how impairment in cognitive domains (attention and information processing, language, visuospatial ability and praxis, learning and memory and executive function) impacts disease management across the care pathway. |
| Jones et al. (2023) (94) | Repeated ED visits | 175,863 PlwD mean 2 LTC | Peer reviewed article, secondary | Quantitative, retrospective analysis of administrative data | Canada | Recurrent ED visits were very common among MLTCiD the history of ED use was the strongest predictor of recurrent use. Groups with highest risk of recurrent ED use also had highest GP use. |
| Kedia et al. (2017) (49) | Health care utilisation | 94,124 older adult patient records (subset 1294 PlwD & cancer) | Peer reviewed article, secondary | Quantitative, retrospective analysis of administrative data | USA | PlwD & cancer diagnoses had higher rates of hospitalizations, hospital readmissions within 30 days, intensive care unit use, and emergency department visits compared with those with dementia only, cancer only, and those with neither condition. PlwD & cancer also had a higher number of primary care visits and specialist visits. |
| Kent et al. (2019) (95) | Repeated ED visits | 24,249 without dementia (mean 2+ LTC), 54,622 PlwD (mean 3+ LTC) | Peer reviewed article, secondary | Quantitative, retrospective analysis of administrative data | USA | Dementia diagnoses were a significant predictor of 30-day ED revisits. |
| Kovaleva et al. (2020) (66) | Integrated Dementia Care | 49 unpaid carers, PlwD (median 5 LTC) | Peer reviewed article, primary | Quantitative, assessment, survey | USA | Carers' health status didn't change significantly during the integrated memory care clinic intervention. MLTCiD reported significantly less severe delusions, depression, and total symptom severity over the 6-month period. |
| Kovaleva et al. (2023) (45) | Transitions of care | 15 carers of PlwD (mean 12.3 LTC including dementia) | Peer reviewed article, primary | Qualitative, interviews | USA | Four categories were identified: 1) the intervention improved carers' understanding of dementia and caregiving; 2) hospitalisation started a 'new level of normal'; 3) PlwD's health concerns; and 4) transitional care intervention development. |
| Kuzmik et al. (2024) (70) | Carer Wellbeing | 431 patient-carer dyads, PlwD and mean 4 LTC | Peer reviewed article, secondary | Quantitative, survey | USA | Care partner burden partially mediated the relationship between patient physical function and care partner anxiety and depression, as well as patient BPSD and care partner anxiety and depression. |
| Lazaroff et al. (2013) (61) | Care pathways | N/A | Peer reviewed article | Commentary | USA | Proposes a framework for organising care around dementia. Understanding the way dementia affects all other conditions is crucial to providing quality care. Care should be organised around dementia, to anticipate crises and decrease spending on ineffective or harmful care. |
| Lee & Neel (2021) (83) | Diagnosis of dementia | 74-year-old woman, 3 LTC and suspected dementia, later assessed | Conference Abstract, primary | Case Study | USA | Case study demonstrates the need for training for ED staff in speciality consultation of geriatric syndromes. |
| Lin et al. (2016) (85) | Diagnosis of dementia | 25,916 PlwD and matched controls, other conditions listed | Conference Abstract | Quantitative, retrospective analysis of administrative data | USA | Many newly-diagnosed patients are readmitted to the hospital within 30 days of discharge, with more costly 30-day readmissions for acute exacerbations of chronic diseases and higher Medicare expenditures than controls during the periods before and following diagnosis. |
| Liu et al. (2023) (118) | Health care utilisation | 10,710 PlwD, 50% 1+ MLTC | Peer reviewed article, secondary | Quantitative, retrospective analysis of administrative data | Taiwan | PlwD & depression no significant emergency visits, significantly less outpatient visits, but higher inpatient visits and longer LOS vs dementia alone. Predictors included age, LTCs, gender, salary-based insurance premiums. |
| Lu et al. (2023) (82) | Hospital readmission | 2075 PlwD (comorbidity index mean 5.7) and 24,053 without dementia | Peer reviewed article, secondary | Quantitative, retrospective analysis of administrative data | USA | Among those with dementia, comorbidity index 3+, or prior ED visits and length of stay were the only significant predictors of hospital readmission. |
| Mitchell et al. (2016) (89) | Hospital outcomes | 8785 PlwD, 23,520 without, 1/3 mean 1+ conditions | Peer reviewed article, secondary | Quantitative, retrospective analysis of administrative data | Australia | MLTCiD less likely to receive hospital-based rehabilitation for hip fracture, but when they do can demonstrate significant functional gain at discharge compared to initial admission. |
| Mustapha et al. (2016) (100) | Coordination of care | 75M, AD +2 LTC | Peer reviewed article, primary | Case study | Singapore | Describes role of a patient navigator to improve coordination of care after hospital discharge. |
| Nguyen et al. (2022) (60) | Integrated Dementia Care | 287 PlwD (comorbidity index mean 9.3) with matched comparator 16 carers | Peer reviewed article, primary and secondary | Mixed methods, retrospective analysis of administrative data, interview | USA | Risk of hospital utilisation was not significantly different between groups, however those in the home care group were more likely to receive palliative care. Caregivers reported coordinated, continuous and convenient care in the home that aligned with their goals, but several unmet needs (e.g. personal care, financial support) were highlighted. |
| Nguyen et al. (2022) (84) | Diagnosis of dementia | 25,278 PlwD | Peer reviewed article, secondary | Quantitative, retrospective analysis of administrative data | USA | Dementias are underdiagnosed in the hospital environment and identified at a later stage than in primary care. |
| Park et al. (2004) (73) | Documentation of Dementia | 597 PlwD (common LTCs listed with %) | Peer reviewed article, secondary | Quantitative, retrospective analysis of administrative data | USA | Identified common nursing diagnoses and interventions given to MLTCiD, discrepancies recorded over who the intervention has been given to (patient/carer). |
| Parke et al. (2013) (78) | Urgent Care | 6 PlwD (conditions documented in text) 11 carers 10 RN 4 nurse practitioners | Peer reviewed article, primary | Qualitative, interview, photographic journal, focus group | Canada | Overarching theme: the way it works: how priorities are determined. 4 negative reinforcing consequences: 1) being under-triaged, 2) waiting: worried about what was wrong, 3) time pressure: lack of attention to basic needs, 4) relationships ad interactions: feeling ignored, forgotten and unimportant. |
| Parker et al. (2020) (28) | Coordination of care | 1861 PlwD 1503 carers | Peer reviewed article, secondary | Systematic Review | Australia | Six elements of care that optimise outcomes: unmet needs; depression; education and support; physical decline; poor quality of life and access and knowledge of community services. |
| Patel et al. (2019) (50) | Coordination of care | 89F, mild dementia and documented MLTC | Conference Abstract | Case Study | UK | Description of discharge planning with using holistic approach for socially isolated patient with repeated admissions. |
| Provencher et al. (2024) (68) | Transitions of care | 7 PlwD, carers, occupational therapists. | Peer reviewed article, primary | Case study | Canada | Risk management during hospital is a dynamic process, involves determining the seriousness and acceptability of risks, reflecting on and actions to manage risks. |
| Rees et al. (2021) (27) | Self-management | N/A | Peer reviewed article, secondary | Systematic Review | UK | Dementia symptoms inhibited self-management of LTC (theme1), while adaptations to routines enabled self-management (theme2). Family carers enabled self-management by proxy when strategies to support self-management were no longer effective (theme 3). HCPs were integral to supporting MLTCiD and carers to manage LTCs (theme 4). |
| Russ et al. (2012) (51) | Diagnosis of dementia | N/A | Peer reviewed article | Commentary | UK | Dementia is underdiagnosed in hospital, MLTCiD are highly vulnerable with many leaving without a diagnosis. Geriatricians have a role to support diagnosis at admission and to assist inpatient care and discharge planning. |
| Sawan et al. (2021) (55) | Medication management | 31 carers | Peer reviewed article, primary | Qualitative, interview | Australia | 3 themes: (a) inadequate information about medication management at discharge; (b) limited engagement in medication management decisions; and (c) difficulties ensuring medication supply post discharge.  Factors that influenced participation in medication management plans at discharge: 1) overwhelmed by discharge processes; 2) proactively seeking information to ensure avoidance of medication harm, 3) Belief in advocacy as part of the role. |
| Scrutton et al. (2016) (77) | Care pathways | N/A | Report | Mixed-methods | UK | Highlights the challenges in prevention, diagnosis, treatment, and management of medical conditions in PlwD. |
| Surr et al. (2020) (47) | Care pathways | 17 PlwD & cancer 22 Carers 19 HCP | Peer reviewed article, primary | Qualitative, ethnography | UK | 4 themes within critical role of supportive networks: 1) Reliance on family support – to access cancer treatment and care; 2) Ability of family to support; 3) The impact of providing support; 4) What if there is no family network? |
| Swidler et al. (2007) (48) | Discharge Planning | 90F, AD (+2 LTCs) | Peer reviewed article, primary | Case Study | USA | Describes ethical, legal issues associated with shared decision making in the unsafe discharge of a patient with repeated, preventable admissions. |
| Tolppanen et al. (2015) (75) | Health care utilisation | 27,948 PlwD (and matched comparators) | Peer reviewed article, secondary | Quantitative, retrospective analysis of administrative data | Finland | Community-dwelling MLTCiD had more admissions to general and specialised inpatient care. Patients had less outpatient visits to general healthcare units. Healthcare costs were generally higher in MLTCiD, roughly translating to an additional $3090/person-year. |
| Turbow et al. (2023) (40) | Hospital Readmission | 36,299 PlwD (mean 3.9 comorbidity index) | Peer reviewed article, secondary | Quantitative, retrospective analysis of administrative data | USA | Fragmented readmissions through different health information exchange systems was associated with readmission. Patients had a higher chance of leaving with home health services when hospitals shared an information exchange system. |
| Umehara et al. (2020) (119) | Hospital readmission | 112 PlwD & HF, 50% 1+LTC | Peer reviewed article, secondary | Quantitative, retrospective analysis of administrative data | Japan | After discharge in patients with HF, presence of dementia influenced readmission within 6 months. Early involvement highlighted as a strategy for lowing readmission rates. |
| Van den Block et al. (2014) (87) | Coordination of care | N/A | Peer reviewed article | Commentary | Belgium | Discusses the need for integration of palliative care early in the dementia pathway, and the need for this to be reflected in key policies concerning dementia care. |
| Verma et al. (2023) (43) | Hospital Readmission | 36,356 PlwD (comorbidity index 4.3) from larger admission cohort | Peer reviewed article, secondary | Quantitative, retrospective analysis of administrative data | USA | Dementia is associated with a significant clinical and financial burden following emergency general surgery, and increased readmission rates. |
| Wang et al. (2014) (58) | Coordination of care | 23 carers of PlwD (MLTCiD documented) | Peer reviewed article, primary | Qualitative, interview | China | The four themes in this study identified three unintended consequences of caregiver practice and identified potential changes to dementia care. Most MLTCiD needed frequent medical treatment and hospitalization, with little coordination between services to prevent acute episodes or reduce number of transitions. Treatment was further compounded by financial strain. |
| Wang et al. (2022) (120) | Hospital readmission | 357,641 PlwD (MLTCs documented) | Peer reviewed article, secondary | Quantitative, retrospective analysis of administrative data | USA | A combination of telehealth post-discharge and enabling services were important interventions in reducing preventable hospitalisations in rural and micropolitan areas. |
| Wood et al. (2021) (88) | Discharge Planning | 269 Dementia + Diabetes (from larger cohort 47.8% 2+ MLTC) | Peer reviewed article, secondary | Quantitative, retrospective analysis of administrative data | Australia | Clinicians considered ageing and frailty, including CI when tailoring treatment regimens for patients with diabetes during discharge planning. |
| Xue et al. (2023) (74) | Hospital readmission | 376 PlwD (comorbidity index M 3.7) from larger cohort | Peer reviewed article, secondary | Quantitative, retrospective analysis of administrative data | USA | Dementia is a significant risk factor for worse long-term outcomes, including readmission and mortality. |
| Zekry et al. (2008) (79) | Hospital outcomes | 151 PlwD (comorbidity index mean 4.9) | Peer reviewed article, primary | Quantitative, surveys, assessments | Switzerland | PlwD had similar levels of MLTC to those without dementia but had poorer functional and nutritional status on admission and discharge. |
| Zuliani et al. (2011) (90) | Hospital readmission | 4466 PlwD (mean 4.7 diagnoses) from larger cohort | Peer reviewed article, secondary | Quantitative, retrospective analysis of administrative data | Italy | PlwD had higher rates of hospital admission and mortality than those without dementia. PlwD had a greater load and different kind of MLTC. |
| Zuo et al. (2022) (52) | Coordination of care | 83M with MLTC, carer for 83F with MLTCiD | Conference Abstract & Poster | Case Study | USA | Describes a case of failure to thrive in patient and carer, with repeated admissions and unidentified carer burnout. |

Abbreviations: AD: Alzheimer’s Disease; ADL: Activities of daily living; BPSD: Behavioural and psychological symptoms of dementia; CI: Cognitive impairment; COPD: Chronic obstructive pulmonary disease; ED: Emergency department; F: Female; GP: General practitioner; HCP: healthcare professional; HF: Heart failure; LOS: Length of Stay; M: Male; MLTCiD: Multiple long-term conditions including dementia; MoCA: Montreal Cognitive Assessment; OTC; Over the counter; PlwD: People living with dementia; RCT: Randomised controlled trial; RN: Registered nurse.

# Appendix 4. CMOCs and illustrative data

| Context-mechanism-outcome configuration (CMOC) | | Illustrative Data Excerpt |
| --- | --- | --- |
| *Theme 1: Dementia care management* | |  |
| CMOC1 | If symptoms of dementia mean patients need more practical support to manage their care needs (C), emotional distress and carer burden are likely to increase (O), because carers feel responsible for more aspects of the patient's care (M). [28, 44, 46-48, 54, 59, 69-74] | *“Relatives perceived CCD to have broader and greater impacts on them than cancer alone would, due to additional difﬁculties with memory, communication, behaviours, and daily activities, with acute, intense care needs associated with cancer potentially tipping the balance of coping” [47]*  *“Role relationship was significantly correlated to FCG strain at all time points” [69]* |
| CMOC2 | If the patient has one or more long-term conditions to manage before the onset of dementia (C), they are less likely to experience difficulties managing their long-term condition after a dementia diagnosis (O), because patient understanding of their long-term condition management is retained (M). [27, 54] | *“The presence of a long-term condition before dementia development was often associated with fewer diﬃculties with self-management of that condition following a dementia diagnosis.” [27]* |
| CMOC3 | If patients or carers believe treatment is not appropriate (C), they will not treat those conditions (O), because their understanding of risk/benefit changes as dementia progresses (M). [46, 61, 75] | *“As dementia progresses, standard treatment options carry an increased risk of adverse outcomes. For example, in a patient with advancing dementia, hypotension may become a greater immediate risk than the consequences of hypertension; in such cases it may be appropriate to gradually increase the blood pressure target. In a patient with diabetes and worsening cognitive impairment, control targets may be relaxed, as the danger of hypoglycemia may outweigh any benefit from tight control. The risks of treatment with drugs such as warfarin (bleeding) or alendronate (esophagitis) increase substantially in the patient who may be unable to take medication competently, making the case against their use unless safe administration can be assured.” [61]* |
| CMOC4 | If carers are supported to advocate for patients with communication difficulties during discharge planning (C), HCPs are more likely to accurately judge the post-discharge support the patient requires (O), because HCPs have a better understanding of the patient’s capabilities (M). [44, 47, 53, 55, 76, 77] | *"Another category was supporting the FCPs [family care partner] to act as advocates, especially in the hospital setting. This included “finding the words” to advocate for the care recipient" to be out of bed and assisted to walk.” [53]*  *“Conflicting realities often led to communication difficulties during transitions. One caregiver, 57, explained how her husband’s view of reality did not align with hers in saying: “…his opinion of what he is capable of and reality is not always the same.” She continued on to express her concerns for safety related to such disparities in perception, indicating that her husband’s descriptions of his own abilities made him seem in need of less support than she deemed necessary. A similar concern was presented by a daughter, 56, in caring for her father, 87. She stated: “…at one point he was on 20 to 22 prescription drugs per day, but you’d ask him, and he’d say, ‘No I don’t take pills.’” [44]*  *These caregivers expressed worry that the health care system was not getting the information required to properly assess the transition needs of the person with dementia.”* |
| CMOC5 | If patients are unable to communicate their care needs to HCPs in hospital (C), their care needs are less likely to be met, increasing risk of harm (O), because HCPs feel pressured, prioritise other patients and forget to attend to patients with dementia (M). [55, 78, 79] | *“Stacy (RN) described an experience with an older man in later stage dementia, who had been chemically sedated because of agitation and forgotten in the midst of a busy department:*  *. . . nobody had thought that he hadn’t eaten in this long. I mean, that’s huge. . .especially with our elderly. Their nutrition is very important . . . he also hadn’t urinated in hours. I mean you should be urinating. You know? Even that like, the fact that nobody had been like, ‘‘Oh my gosh, maybe let’s at least start in IV in this man so he can get some ﬂuids.’’ We are neglecting so much of our elderly and then elderly with dementia is even just one more step, that’s one more barrier for us. ‘Cause they can’t tell us what they need, they don’t have a voice for themselves and we can’t always be that advocate for them because we’re too busy.” [78]* |
| CMOC6 | If patients and carers are provided with tailored and concise education about dementia, care management and social needs (C), carers and patients will be better prepared to support care needs post-discharge, reducing carer burden (O), because understanding of care needs is increased, reducing carers stress, and increasing confidence in their ability to act (M). [28, 45, 61] | *“Therefore, a transitional care intervention may also address aspects that are not directly pertinent to the transitional period. For example, insurance and legal issues may not be directly related to the prevention of post-hospitalization complications and readmissions. Yet, because caregivers ascribe much stress to these care aspects and, in their descriptions of hospitalization and post-discharge care, refer to these topics frequently, it is necessary to provide education and support in these areas.” [45]* |
| CMOC7 | If community support for patients is not inclusive of MLTCiD (C), patients and carers are less likely to engage with them (O), because they don’t think the groups understand or accommodate their needs (M). [47] | *“that's another [cancer speciﬁc] group that we tried once a month. But then I was so tired after looking after the children and I … couldn't face just going out again. … so I dropped him off on his own. It didn't work for him. … Being deaf, … speakers are … not used to projecting their voices. Sometimes [when they are] talking … he'd no way of getting it [understanding the discussion], and so after a couple of times going on his own he just said, ‘I don’t think I want to go anymore’. [(Interview daughter CC009)]” [47]* |
| *Theme 2: (HCP) Knowledge* | |  |
| CMOC8 | If HCPs in hospitals are not knowledgeable of the signs and symptoms of dementia (C), they will be less likely to diagnose patients with dementia, limiting the suitability of their discharge planning (O), because HCPs’ have limited awareness of dementia, and they don’t feel confident in their ability to identify patients (M). [50, 51, 59, 80-83] | *“Every individual admitted to hospital receives an admission assessment including documentation of past medical history, a systems enquiry and physical examination to ensure relevant and co-existing pathology is not missed. Cognitive screening, analogous to the physical examination, is particularly important in de novo detection of dementia and indeed is universally recommended for all inpatients aged over 65 [8]. Yet the 2011 National Audit of Dementia Care in General Hospitals showed that having a relevant policy did not correlate with actual practice, for example 75% of hospitals advised mental state assessments but only 43% of casenotes examined had evidence of this being carried out [9]. This means that in the UK alone tens of thousands of people with undiagnosed dementia are admitted to and discharged from general hospitals without any cognitive testing or other cognition-speciﬁc assessments, with the consequence that their dementia remains undetected and untreated.” [51]* |
| CMOC9 | If HCPs are educated about the impact of symptoms of dementia on long-term condition management (C), patients diagnosed with dementia in hospital are more likely to receive discharge planning that meets their care needs (O), because HCPs understand how care for people with dementia should be managed, increasing confidence in their ability to plan post-discharge care (M). [46, 51, 54, 73, 74, 77, 84-86] | *“The evidence suggests that staff at all levels, including more senior staff, need appropriate training on dementia. Some training may need to be tailored to specific conditions, for example identifying the best strategies for the rehabilitation of PLWD who have had a stroke.” [54]* |
| CMOC10 | If HCPs are not knowledgeable of the benefits of palliative care for patients with MLTCiD (C), they will be less likely to make timely referrals to palliative care, where patients may avoid unnecessary transitions(O), because they aren’t aware that dementia is suitable for palliative care (M). [60, 65, 84, 87] | *“That no signiﬁcant thematic differences were identiﬁed between caregivers of patients with and without dementia suggests that differences in utilization of hospice services may not be due to differences in patients’ needs and preferences, but rather supports the theory that healthcare providers may not recognize dementia as a terminal illness and therefore may not refer patients for these services in a timely manner.” [65]* |
| CMOC11 | If medication management for patients with dementia is informed by the knowledge of their other long-term conditions (C), hospitalisations for acute exacerbations of other conditions can be prevented (O), because HCPs understand the interaction between MLTCiD and the patient’s care needs (M). [54, 88] | *“And some of the nursing staff have been working with their community colleagues quite intensively to develop individualised regimes to try and, you know, if they eat half their meal to give this amount of insulin, if they eat all of the meal to give a different amount of insulin, so it’s almost like a sliding scale, it’s individualised, it’s not for every patient but for those who sometimes refuse food it’s sometimes really quite a helpful way to prevent them having to keep coming back in with hypos or highs. Diabetes consultant, diabetes focus group, south-east” [54]* |
| CMOC12 | If HCPs decision making process are informed by their knowledge of dementia (C), patients access to diagnoses, treatment or care management support are likely to be reduced (O), because HCPs perceive patients with dementia as being less able to benefit (M). [50, 54, 89, 90] | *“We also run a huge sub-management programme which does have a module for people who have had strokes as well as two general modules and somebody with dementia we wouldn’t invite necessarily to those modules so they themselves would miss out on a huge bit of secondary intervention and advice that they might get otherwise and because we don’t invite the carers automatically the carer also doesn’t get the secondary intervention advice. Physiotherapist, stroke focus group, south-east” [54]* |
| CMOC13 | If HCPs believe patients with dementia present to the ED with social/non-urgent needs (C), patients are more likely to be under-triaged, increasing their risk of harm & readmission (O), because HCPs feel pressured to prioritise treating urgent medical needs and don't feel responsible for other care needs (M). [48, 50, 57, 61, 77, 78, 83, 91, 94, 95] | *“The hospital focus on minimising patient length of stay and achieving occupancy targets meant that the clinical team were disproportionately focused on discharge-planning. The nurses in this study reported that the allied health staff spent more time admitting and discharging patients than providing therapy. The focus is on turnover – get ‘em in get ‘em out, but these patients [with dementia] need a lot of attention. . . We have OT and we have physio and everyone does a good job but there could be a bit more not just concentrating on discharge planning but a better time while they’re here in the ward. (Mandy, RN)” [91]*  *“Rachel (RN) explained: ‘‘So . . . like who’s the priority? That dementia patient may not be a priority because we have somebody who’s having an MI (myocardial infarction) right now or actively seizing’’. Sara (RN) commented ‘‘the focus of the Emergency Department is to ﬁnd today’s problem, ﬁx it and send them home.’’ Participating nurses distinguished between patients with and without urgent physiological problems. This suggested that if the older adult with dementia had an obvious urgent physiological problem, their priority for care would increase. However, if their presenting problem was not obvious, they were at risk for being under-triaged.” [78]* |
| *Theme 3: (HCP) Standards* | |  |
| CMOC14 | If there is no standardised approach to recording and reporting dementia diagnoses during transitions between HCPs (C), HCPs are less likely to be aware of dementia diagnoses (O), because they prioritise their own work area (M). [46, 51, 54, 67, 80, 84, 90] | *“Staﬀ was not always aware of dementia, often needing to proactively search medical records to establish a dementia diagnosis, which was not always recorded and was seldom documented in an easily accessible and consistent way.” [46]* |
| CMOC15 | If patient-held records to transfer dementia diagnosis information are not a priority in the healthcare system (C), patient-held records will not be developed for patients to use (O), HCPs don't see the value of developing patient-held records to support transfer of information, so don't prioritise this (M). [54] | *“However, although hand-held personalised documents such as the ‘This is me’ passports exist as a way of ensuring that PLWD can share key information about themselves with HCPs [see http://alzheimers.org.uk/ thisisme (accessed 7 January 2016)], we found that very few PLWD, carers or HCPs were aware of them. One carer thought that she had been given a ‘This is me’ passport but said that it wasn’t filled in and she didn’t use it. One patient mentioned having had a small card that detailed that he had Alzheimer’s disease and his next of kin, medication and doctor’s details, but he had misplaced it.” [54]* |
| CMOC16 | If documentation doesn't specify whether interventions are delivered to patients or carers in hospital (C), this information is less likely to be transferred correctly between HCPs, and carers will be less likely to receive educational interventions (O), because other HCPs assume patients have received the intervention (M). [73] | *“In the behavioural area, teaching interventions showed much higher frequencies compared with other interventions such as behaviour therapy, cognitive therapy and communication. As mentioned previously, considering the inability of people with dementia to process information, this intervention should be directed toward family caregivers and the target of the intervention needs to be documented clearly” [73]* |
| *Theme 4: (HCP) System* | |  |
| CMOC17 | IF emergency department environments are distressing to patients (C), carer burden is likely to increase (O), because they feel responsible for keeping the patient calm (M). [55, 78] | *“Some caregivers reported that the hospitalization caused caregivers' tiredness and stress from managing the emotional needs of the person with dementia and addressing exacerbation of symptoms triggered by the unfamiliar hospital environment.” [55]* |
| CMOC18 | If emergency department environments are not dementia-friendly and exacerbates behavioural and psychological symptoms of dementia (C), the patient’s presenting care needs are likely to be masked (O), because HCPs assume increased confusion is due to the dementia and don't recognise underlying care needs (M). [46, 73, 78, 94] | *“Our ﬁndings indicate that nursing triage assessment did not take into account atypical presentation of a dementing illness or the impact of the chaotic atmosphere of the ED on an older person’s ability to adapt.” [78]* |
| CMOC19 | If discharge planning for patients with complex needs requires collaboration from multiple HCPs (C), patients with dementia are more likely to experience delayed discharge (O), because collaboration to manage multiple care needs is not a priority in the healthcare system (M). [41, 43, 75, 85, 89, 92, 93] | *“The key points are that the poor physical health of patients, low cognitive capacity and complex medical treatment can potentially slow the discharge process. This is because of the need to involve a wide range of professionals in the care of patients with complex needs.” [93]* |
| CMOC20 | If post-discharge support for dementia does not fit with existing care pathways (C), patients are more likely to be discharged without appropriate care (O), because HCPs don't feel responsible for organising social care (M). [44, 46, 47, 54, 55, 81, 91, 96-98] | *“Additionally the clinical team concentrated almost exclusively on treating the admission diagnosis of the patients rather than considering the patients’ situation as a whole. In some instances this was problematic, especially for patients with a number of other health conditions and social problems such as the 100 year-old visually impaired woman referred to in the quote below:*  *[You seem to have quite a lot of patients with social complex problems, VCAT guardianship applications and so forth] More and more. And now because of the budgetary constraints I guess well that’s just not our problem· · ·If they’re going home . . . like with [patient] . . . there’s no-one to supervise her, an outside toilet, no heating, leaking roof, it’s not our problem because physically she’s OK. Yeah so that’s hard to watch when you see some people going home that you think wow, that’s just a nightmare waiting to happen. . . (Pam, EN)” [91]* |
| CMOC21 | If there are no care pathways to support patients with MLTCiD (C), family and friends will spend more time supporting the patient (O), because they feel responsible for the patient’s welfare (M). [54] | *“Unpaid care was common, with over two-thirds of people with dementia alone reporting use of informal help from family and friends. Use of unpaid care was even higher for individuals with dementia and one of the target health conditions, with around eight out of 10 people reporting informal help*  *Our study suggests that care pathways need to recognise the contribution of family members and, when available, incorporate their contribution in the care of PLWD.” [54]* |
| CMOC22 | If patients with dementia do not have a support network to help manage their care needs during transitions between care services (C), they are less able to navigate healthcare systems and receive treatment (O), because HCPs do not feel responsible for organising the patient's care (M). [27, 46, 47, 54] | *“The model [triangle of care] requires further testing and development, specifically on how it is implemented and how it can address issues such as confidentiality, the involvement of multiple carers or divergence of opinion between the PLWD and the carer. It also cannot address the needs of someone whose carer network involves loose ties or shifting patterns of unpaid support.” [54]*  *“There is no one to support this sort of thing ... there was some memory nurse input, so we requested that they provided some support around escorting. But again, they didn’t seem to see it was their role. Social worker (L0013)” [46]* |
| CMOC23 | If there is a designated HCP to facilitate early follow-up with the patient after discharge (C), patient safety during the transition is improved (O), because the patient is less confused and knows who to go to for support (M). [54, 56, 61] | *Amedisys has placed CTCs in the acute care facilities that it serves. The CTC’s responsibility is to ensure that patients transition safely home from the acute care setting. With fragmentation of care, patients are most vulnerable during the initial few days postdischarge; this is particularly true for the frail elderly. Consequently, the CTC meets with the patient and caregiver as soon as possible upon his or her referral to Amedisys to plan the transition home from the facility and determine the resources needed once home. [56]* |
| CMOC24 | If care after hospital discharge is holistic, and centred around the patient's dementia (C), MLTCs can be managed alongside dementia (O), because HCPs can understand how the interplay of MLTC-MiD influences care needs (M). [61, 66, 77, 78, 99] | *People with dementia are being underdiagnosed with comorbidities and, when diagnosed, receiving poorer management and treatment of these conditions, in part because of a failure by the health system to recognise the individual as a whole and instead focussing on the person as a patient with a given diagnosis. Many clinicians are familiar with the gallows humour of the surgeon who reported “the operation was a success but the patient died,” but they would perhaps find this less amusing if it was changed to “the glucose was well controlled but the older adult ended up in the intensive care unit on mechanical ventilation in a hypoglycaemic coma69”. [77]* |
| *Theme 5: The role of family/friends* | |  |
| CMOC25 | If family members feel they are expected to care for the patient (C), they will accept the role of a carer (O), because they feel as though they have no choice, and are responsible for the patient’s welfare (M). [41, 47] | *“However, for more distant relatives this could entail an unexpected and perhaps unwelcome obligation:*  *And I think the cousin felt a little bit like, I suppose it's all down to her. That's a lot of responsibility for her to deal with, as a cousin. As a daughter or a son, it's sort of expected, isn't it? But as a cousin, it's different. [(Interview Lung Clinical Nurse Specialist SL003)]” [47]* |
| CMOC26 | If patients are discharged home and decline paid carers (C), family members will accept more caring tasks (O), because they feel responsible for the patient (M). [45] | *“When PLWD discharged directly home, sometimes it meant starting team caregiving or hiring assistants. Son-033 described how his mother “hit the ceiling” when he announced about paid caregivers. Hence, he negotiated caregiving schedule with his sister.” [45]* |
| CMOC27 | If patients are discharged with the assumption that carers will manage their additional medical needs at home (C), carer emotional distress is likely to increase, as does patient risk of harm if carers are not capable (O), because carers feel unsupported and unprepared to assist medical care needs (M). [45, 47, 48, 54, 68] | *“It was also recognised that family carers of people with dementia and diabetes often take on responsibility for giving medication, managing diet, etc., and that they may need additional education and support to equip them for this role.” [54]*  *“there was an expectation by oncology staff that relatives would be willing and able to deliver intimate care tasks; in this case, that a wife would give an enema to her husband prior to prostate radiotherapy: ‘I was just thinking, I don't think he'd be able to do an enema himself with having dementia’ says CL0036 [wife of PL0035 with prostate cancer and dementia]. The doctor replies to CL0036 ‘You'll be there’ [(Field notes from observations of participants PL0035 and CL0036)]” [47]* |
| CMOC28 | If family members have conflicting opinions on the care the patient should receive (C), the main carer will struggle to make decisions on the patient's behalf (O), because of increased uncertainty (M). [47, 100] | *“There's a lot to think about and I got really stressed with it, because I thought, everyone will want an input, because I've got family and I have to tell them and they might push to say, well she should have the operation, … But suddenly when you've got family, everyone has got an opinion, but they don't know the whole picture. [(Interview carer daughter CL0011)]” [47]* |
| CMOC29 | If the patient is no longer able to report their symptoms of ill health (C), carer stress is likely to increase, patients may not receive appropriate care (O), because carers feel increased uncertainty and fear of missing symptoms (M). [27] | *“Communication diﬃculties often impaired a person with dementia’s ability to report symptoms to family carers or healthcare professionals, and this could inhibit long-term condition management. Family carers reported guessing what symptoms the care recipients were experiencing due to their inability to report symptoms. ‘…I want to do the right thing by him, but he has not been able to tell me when he is ill or hurting for over a year now’ ( Sadak et al., 2017 ; Family Carer ).” [27]* |
| CMOC30 | If transitional care interventions address the mental health and wellbeing needs of carers (C), carer engagement in the intervention increases, mental health and wellbeing are likely to improve (O), because they feel supported, increasing feelings of hope and self-efficacy (M). [28, 45, 53, 64, 69, 101, 102] | *“The main findings of this study were a statistically significant difference between the users and nonusers of MT4C with regard to general self-efficacy (the confidence in their ability to deal with difficult situations). General self-efficacy significantly increased in the user group.” [64]*  *“It was important to further examine differences between users and nonusers in the treatment group, as the finding that self-efficacy was higher in users versus nonusers supports the intervention model in which MT4C has the potential to increase quality of life by increasing self-efficacy.” [64]* |

# Appendix 5. Supplementary reference list

116. Dooley J, Booker M, Barnes R, Xanthopoulou P. Urgent care for patients with dementia: a scoping review of associated factors and stakeholder experiences. BMJ Open. 2020;10(9):e037673.

117. Henkle BE, Freese RL, Dahlheimer M, Kane C, Hoth KF, Kunisaki KM. Cognitive function and inhaler technique following recovery from exacerbations of COPD. ERJ Open Research. 2023;9(3).

118. Liu CC, Liu CH, Wang JY, Chang KC. Health-care utilization among dementia patients with or without comorbid depression in Taiwan: A nationwide population-based longitudinal study. International Journal of Geriatric Psychiatry. 2023;38(2):e5889.

119. Umehara T, Katayama N, Tsunematsu M, Kakehashi M. Factors affecting hospital readmission heart failure patients in Japan: a multicenter retrospective cohort study. Heart Vessels. 2020;35(3):367-75.

120. Wang N, Buchongo P, Chen J. Rural and urban disparities in potentially preventable hospitalizations among US patients with Alzheimer's Disease and Related Dementias: Evidence of hospital-based telehealth and enabling services. Preventive Medicine. 2022;163:107223.
